# Fast emission color switching of circularly polarized luminescence in platinum(ii) liquid crystalline co-assembly†

**DOI:** 10.1039/d5sc02285a

**Published:** 2025-05-13

**Authors:** Guo Zou, Qihuan Li, Zhenhao Jiang, Wentong Gao, Yixiang Cheng

**Affiliations:** a State Key Laboratory of Analytical Chemistry for Life Science, School of Chemistry and Chemical Engineering, Nanjing University Nanjing 210023 P. R. China yxcheng@nju.edu.cn; b School of Materials Science and Engineering, Nanjing Institute of Technology Nanjing 211167 P. R. China

## Abstract

Developing stimuli-responsive circularly polarized luminescence (CPL) materials that feature fast emission color switching for advanced information encryption presents a scientifically significant yet formidable challenge. Herein, we construct a supramolecular co-assembly system demonstrating transiently responsive CPL emission color switching, enabling mechanically-modulated information encryption. Combining a highly luminescent Pt(II) liquid crystal (Pt8) with the anchored binaphthyl inducers (*R*/*S*-M) forms chiral co-assemblies (*R*/*S*-M)_0.03_-(Pt8)_0.97_, which assemble into twisted nanobelts (180 °C) and helical nanofibers (260 °C) exhibiting green (*λ*_em_ = 545 nm, *g*_em_ = 0.038) and red CPL (*λ*_em_ = 640 nm, *g*_em_ = 0.133), respectively. Notably, mechanical grinding transforms the 180 °C-annealed (*R*/*S*-M)_0.03_-(Pt8)_0.97_ into nanoparticles, resulting in a fast dynamic switching of CPL emission color from green to orange-red (*λ*_em_: 545 → 625 nm, *g*_em_: 0.038 → 0.058). Reheating the grinding films (*R*/*S*-M)_0.03_-(Pt8)_0.97_ to 180 °C restores the initial green CPL of the nanobelts. Based on the fast CPL emission color switching, we demonstrate the applications of these supramolecular chiral co-assemblies for mechanically-modulated information encryption.

## Abstract 2

Transiently responsive co-assemblies (*R*/*S*-M)_0.03_-(Pt8)_0.97_ are developed by combining achiral complex Pt8 and chiral inducers *R*/*S*-M, enabling fast emission color switching of circularly polarized luminescence and unique information encryption.

## Graphical abstract

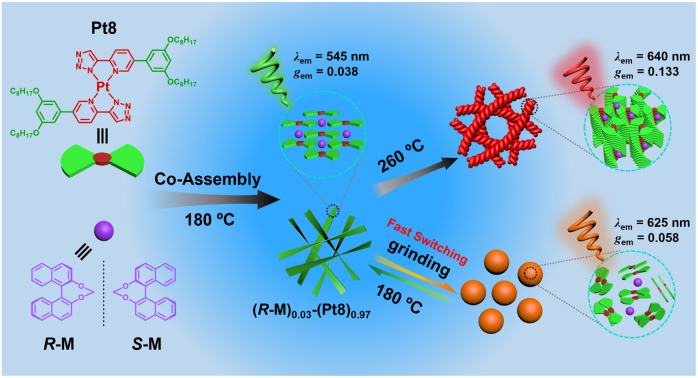

## Introduction

Circularly polarized luminescence (CPL) has garnered significant attention in recent decades due to its promising applications in information encryption,^1^ optical anticounterfeiting,^2^ three-dimensional displays,^3^ chiral polymerization,^4^ and smart sensors.^5^ The wavelength tunability, variable emission dissymmetry factor (*g*_em_) values and sign inversion of stimuli-responsive CPL materials enable them to exhibit a more flexible and deeper optical information dimension compared to conventional luminophores.^1*a*,6^ Stimuli-responsive multicomponent co-assembled CPL materials are particularly noteworthy for their efficient construction, excellent processability and easy derivatization.^7^ Based on the non-covalent interactions during the co-assembly process, chiral supramolecular co-assembly systems offer various protocols to regulate CPL behaviors through external stimuli such as solvent,^8^ temperature,^9^ mechanical force,^10^ pH,^11^ light,^1*b*,12^ metal ions,^7*c*^ electric fields^13^ and magnetic field.^14^ For instance, Liu and co-workers revealed that co-assembled L-PAG/β-DCS can form nanofibers with blue CPL signals and nanotube structures with green CPL emissions upon different temperature control of slow and fast cooling processes, respectively.^6*a*^ However, mechanically-responsive CPL materials have been less studied, despite their environmentally friendly, solvent-free and clean processing virtues. Mechanically-responsive CPL materials exhibit ultrafast response characteristics, achieving actuation within millisecond to microsecond timescales, which significantly outperforms conventional stimuli-responsive CPL materials such as thermo-, chemo- and photo-responsive CPL systems.^10*a*,15^ Yamashita *et al.* proved that the CPL signals of a non-chiral hydrogel embedded with rhodamine B can be detected upon mechanical stirring and exhibit sign inversion when the stirring direction was changed.^16^ To the best of our knowledge, most of the currently reported mechanically-responsive CPL materials are limited to achieving CPL on/off switching and have not demonstrated the capability for CPL emission color switching.^5*a*,10*b*,13,16,17^

As soft materials, Pt(II) liquid crystals exhibit high sensitivity to chiral group perturbations and realize strong CPL-activity *via* intermolecular Pt⋯Pt and π–π stacking interactions in chiral assembly processes.^18^ Our group has developed highly CPL-active helical nanofibers by combining an achiral robot-like Pt(II) liquid crystal with the anchored chiral binaphthyl inducer (*λ* = 642 nm, *g*_em_ = 0.27).^18*a*^ And very recently, we have constructed efficient helical columnar emitters of chiral homoleptic Pt(ii) liquid crystals, which were utilized to fabricate high-performance solution-processed circularly polarized electroluminescence devices.^18*b*^ On the other hand, Pt(ii) liquid crystals have rich charge transfer transitions and are very sensitive to external stimuli.^19^ Upon mechanical grinding, Pt(ii) liquid crystals tend to form intermolecular Pt⋯Pt contacts, generating a strong redshifted emission. Conversely, when volatile organic compounds (VOCs) are adsorbed, the initial emission of monomers can be recovered *via* breaking the intermolecular Pt⋯Pt contacts. Swager and co-workers have demonstrated that Pt(II) liquid crystals display reversible mechanically-responsive emission transformations under the processes of mechanical grinding and solvent adsorption.^20^ Therefore, through rationally controlling Pt⋯Pt interactions, Pt(ii) liquid crystals can serve as potential mechanically-modulated CPL luminophores to ensure security and complexity in information encryption and optical anticounterfeiting.^19*e*,21^

**Scheme 1:**
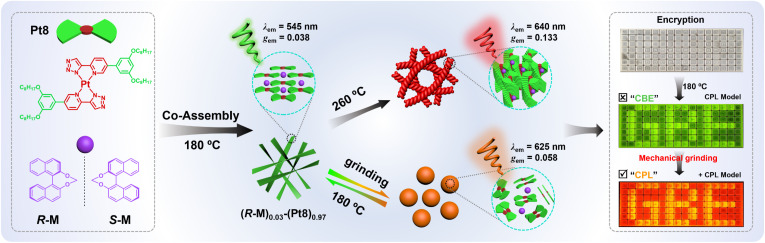
The schematic diagram of dynamic reversible CPL color switching in supramolecular Pt(ii)-based co-assembly for mechanically-modulated information encryption.

Herein, we construct mechanically-modulated CPL co-assemblies *via* combining an achiral homoleptic Pt(ii) liquid crystal (Pt8) with the chiral binaphthyl inducer (*R*/*S*-M) (Scheme 1). The achiral Pt8 is capable of not only serving as an efficient mechanically-responsive luminophore, but also provides a liquid crystal (LC) media that is highly sensitive to chiral group perturbations. The anchored binaphthyl derivative *R*/*S*-M is employed as a chiral inducer due to its excellent chirality induction ability and negligible influence on the photoluminescence (PL) behavior of a luminophore.^9,22^ The resultant chiral co-assembly (*R*/*S*-M)_0.03_-(Pt8)_0.97_ is distinctly temperature-responsive in CPL at 180 °C (*λ*_em_ = 545 nm, |*g*_em_| = 0.038) and 260 °C (*λ*_em_ = 640 nm, |*g*_em_| = 0.133). More intriguingly, the 180 °C-annealed films (*R*/*S*-M)_0.03_-(Pt8)_0.97_ exhibit fast mechanically-modulated CPL emission color switching from green to orange-red (*λ*_em_: 545 → 625 nm, *g*_em_: 0.038 → 0.058). To our knowledge, this study represents the inaugural demonstration of mechanically-modulated chiral co-assemblies that exhibit transiently responsive CPL emission color switching. Moreover, utilizing these mechanically-modulated CPL-active materials, we successfully implement mechanically-modulated information encryption and optical anticounterfeiting.

## Results and discussion

### Synthesis and characterization

The achiral homoleptic Pt(ii) liquid crystal Pt8 was synthesized in 94% yield *via* the reaction of K_2_PtCl_4_ with *N*^*N*-cyclometalated ligand A-7 (Scheme S1†). The free ligand A-7 was prepared using a series of Sonogashira,^23^ Suzuki–Miyaura cross-coupling^24^ and Sharpless copper-catalyzed Huisgen 1,3-dipolar cycloaddition protocols,^25^*etc*. This approach eliminates the need for the redundant synthesis of chloride containing organoplatinum(ii) precursors.^26^ The anchored chiral binaphthyl inducers *R*/*S*-M were synthesized according to previous methods.^9,22^ Detailed synthesis and characterization data of Pt8, including ^1^H and ^13^C NMR and MALDI-TOF MS data, are provided in the ESI.† The targeted Pt(ii) complex was further purified *via* triple recrystallization in a mixture of chloroform and acetone, followed by column chromatography before investigation.

### Temperature- and mechanically-responsive PL properties and mesophase behaviors of Pt8

**Fig. 1:**
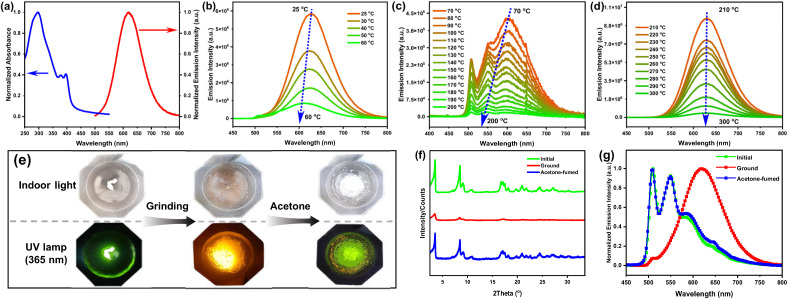
Ultraviolet-visible (UV-vis) absorption and PL spectra of Pt8 in a spin-coated film (6 mg mL^−1^ in chloroform; 1000 rpm, 30 s) at room temperature (a); PL spectra of Pt8 after thermal annealing at different temperatures (b–d); the photos (e), XRD patterns (f) and PL switching (g) of Pt8 at the initial powder, ground and acetone fumigation states.

In chloroform, Pt8 exhibits low-energy absorbance at 350–400 nm (Fig. S1a†), originating from metal-to-ligand charge transfer (MLCT) and intraligand charge transfer (ILCT) transitions, corroborated by the absorbance of free ligand A-7 (Fig. S2†) and time-dependent density functional theory (TDDFT) calculations (Fig. S3, S4 and Table S1†). In the spin-coated film, Pt8 exhibits a newly generated absorption tail from 400–550 nm (Fig. 1a), suggestive of intermolecular Pt⋯Pt and π–π stacking interactions.^18*b*^ Upon excitation at 365 nm, spin-coated film Pt8 exhibits a more intense orange-red emission (*λ* = 620 nm; *Φ* = 82%; *τ* = 5.73 μs) (Fig. 1a and S5†) compared to that in chloroform (*λ* = 409 nm, *Φ* = 2.5%, *τ* = 2.08 *n*s) (Fig. S1†).

Pt8 exhibits temperature-responsive PL properties after thermal annealing at different temperatures (Fig. 1b–d and S6†). Specifically, orange-red metal–metal-to-ligand charge transfer (MMLCT) emissions (*λ*_em_ = 620 nm) and a decrease in PL intensity can be observed in the spin-coated film after thermal annealing at 25–60 °C (Fig. 1b). Upon increasing annealing temperature to 70–200 °C, the PL spectra exhibit an obvious blue shift to the green region (Fig. 1c). The blue-shifted spectral profiles may be rationalized by the thermal perturbation that disrupts the formation of an excimer.^27^ Conversely, after annealing at higher temperatures (210–300 °C) (Fig. 1d), the orange-red emissions of Pt8 reappeared at 628 nm, which is assigned to the reconstruction of excimers in long-range ordered liquid crystal superstructures. In addition, the red-shifted absorption tail of Pt8 becomes more obvious at higher annealing temperatures (Fig. S7†), stemming from stronger intermolecular Pt⋯Pt and π–π stacking interactions.^28^

After grinding with a pestle, a drastic color change from white to brown of Pt8 is easily captured by the naked eye under indoor light (Fig. 1e), indicating that the pristine sample is sensitive to mechanical force stimuli. As depicted in Fig. 1f, the initial Pt8 demonstrated many sharp diffractions in small and wide-angle regions. After grinding, the number and intensity of the X-ray diffractogram (XRD) peaks both decreased, due to a collapse of the crystal structure in the ground state, indicating that the ordered molecular arrangement of the initial powder became more amorphous upon mechanical grinding. Under UV irradiation (*λ* = 365 nm), the emissions of Pt8 changed sharply from green (*λ*_em_ = 509 nm, *Φ* = 30%) to orange-red (*λ*_em_ = 620 nm, *Φ* = 85%) after mechanical grinding (Fig. 1g), showing a wide chromic shift response of 111 nm. And the orange-red emissions originate from the MMLCT transitions that are associated with intermolecular Pt⋯Pt interactions. Moreover, the green emissions can be recovered by the addition of acetone (Video S1†). These results demonstrate that Pt8 is an efficient mechanically-responsive luminescent material, which may hold promise as a mechanically-responsive luminophore for designing and constructing stimuli-responsive CPL materials.

**Fig. 2:**
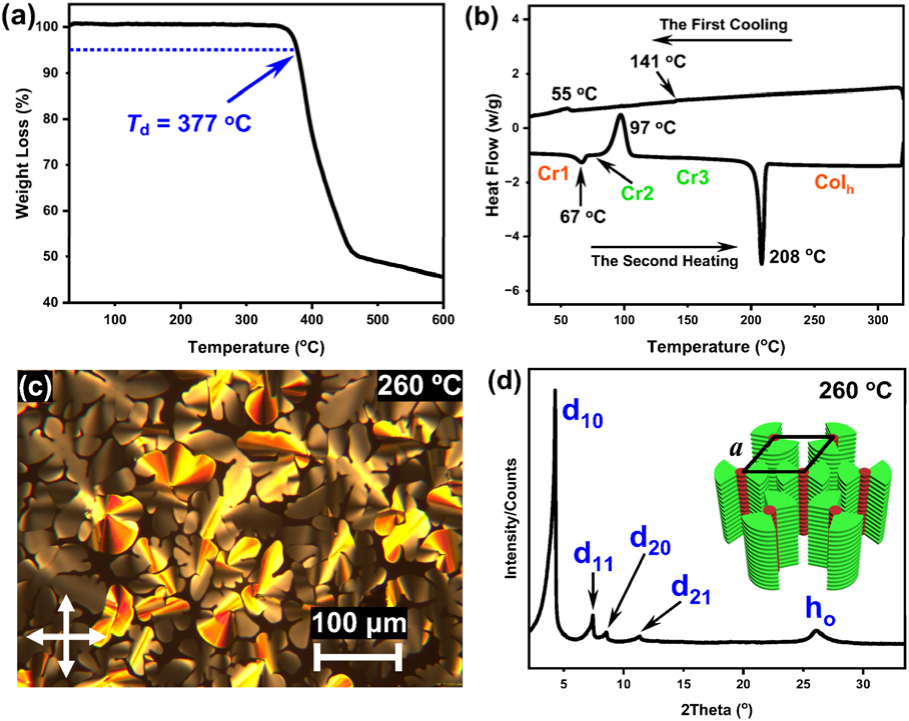
TGA curve (a) and DSC traces (b) of Pt8 (at a scan rate of 10 °C min^−1^ under a nitrogen atmosphere); POM image (c) and XRD pattern (d) of Pt8 at 260 °C on cooling.

Pt8 exhibits excellent thermal stability with a degradation temperature of 377 °C, as reflected by thermogravimetric analysis (TGA) (Fig. 2a). Differential scanning calorimetry (DSC) experiments conducted within the temperature range of 25–320 °C (Fig. 2b) indicated the enantiotropic liquid crystal behavior of Pt8. The DSC traces reveal a high-enthalpy endothermic peak at 208 °C on heating, corresponding to the phase transition from a crystalline to liquid crystalline state. Moreover, an exothermic crystallization peak was also observed at 97 °C on heating, indicative of the crystalline transformations from crystal 2 (Cr2) to Cr3. We observed the dendritic textures at 260 °C in polarized optical microscopy (POM) measurements, corresponding to hexagonal columnar phases (Col_h_) (Fig. 2c). Notably, the clear point temperature of Pt8 is as high as 365 °C (Fig. S8†), indicating that Pt8 possesses ultra-high thermally stable mesophase structures. Variable-temperature XRD analyses display characteristic patterns of the Col_h_ phase upon cooling (Fig. S9†). Herein, the pattern of Pt8 at 260 °C was further extracted (Fig. 2d), showing four distinct diffractions in the small-angle region with 2*θ* of 4.28°, 7.43°, 8.55° and 11.37°. The corresponding *d* spacings are 20.66 Å, 11.92 Å, 10.36 Å and 7.80 Å, respectively, which are well consistent with the ratio of 1 : 1/√3 : 1/2 : 1/√7. These peaks are indexed to *d*_10_, *d*_11_, *d*_20_ and *d*_21_ reflections. The lattice constant (*a*) of Pt8 was calculated to be 23.86 Å. Significantly, a broad diffuse peak (*h*_0_) of *d* = 3.42 Å was observed at 2*θ* = 26.08°, attributed to the intermolecular π–π stacking interactions along the column. These results indicated that Pt8 is a highly thermally stable Col_h_ liquid crystal, which could serve as an ideal matrix for constructing efficient stimuli-responsive co-assembled CPL materials.

### Photophysical properties of chiral co-assemblies *R*-M-Pt8

**Fig. 3:**
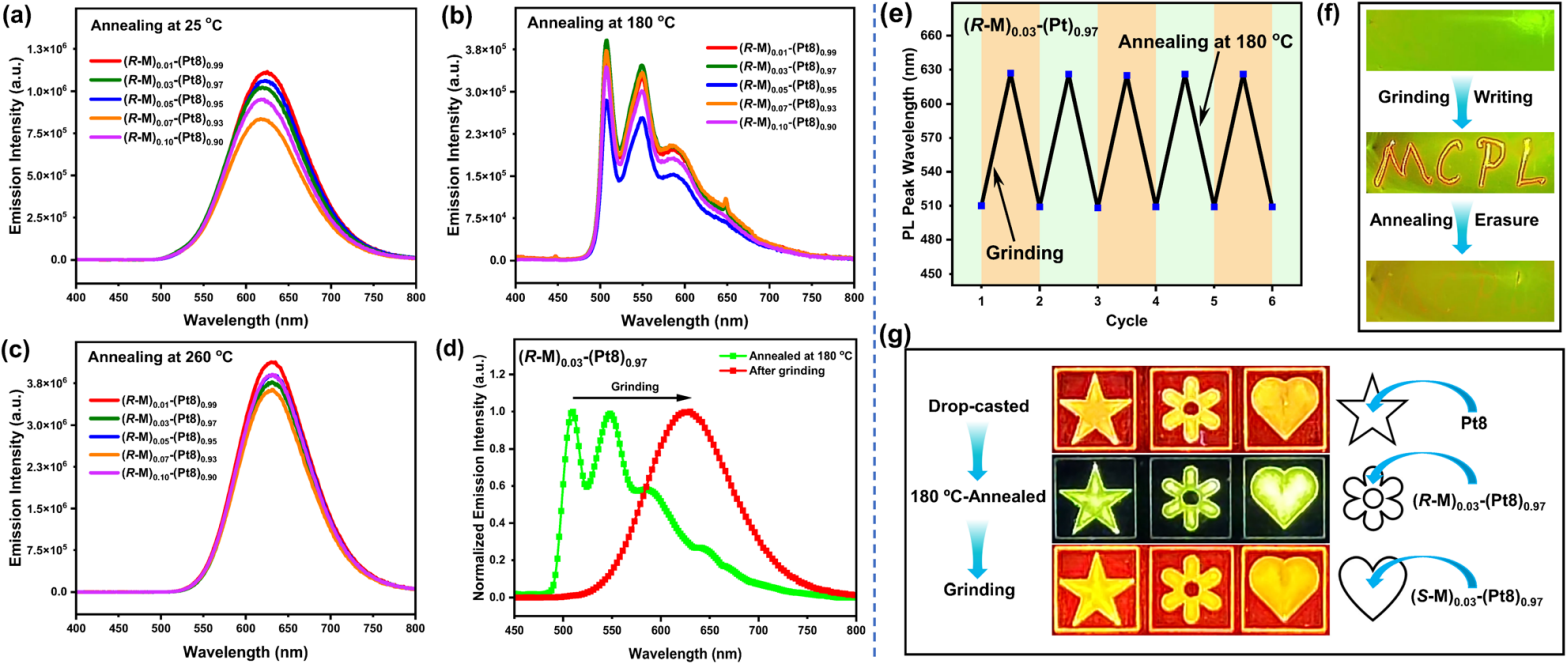
PL spectra of chiral co-assemblies *R*-M-Pt8 at different annealing temperatures (a–c); PL spectra of the 180 °C-annealed film (*R*-M)_0.03_-(Pt8)_0.97_ after grinding (d); reversible process of the mechanochromic PL switch of 180 °C-annealed (*R*-M)_0.03_-(Pt8)_0.97_ (e) (monitored at maximum emission wavelength); fast writing and erasure applications of (*R*-M)_0.03_-(Pt8)_0.97_ (f); optical anticounterfeiting applications of Pt8 and chiral co-assemblies (*R*/*S*-M)_0.03_-(Pt8)_0.97_ (g).

We prepared chiral co-assemblies by first dissolving 6 mg each of *R*/*S*-M and Pt8 in 1 mL of chloroform. Subsequently, mixed solutions for the chiral co-assembly were prepared by adding *R*/*S*-M solutions to the Pt8 at different molar ratios of chiral inducer (1–10%). Finally, blend films were spin-coated onto 1 cm × 3 cm quartz plates from the chloroform solutions at 1000 rpm (30 s), and then annealed at 180 and 260 °C for 15 minutes, respectively. The UV-vis absorption and PL spectra of the chiral co-assemblies *R*-M-Pt8 were investigated and showed similar absorptions, which are consistent with Pt8 before and after thermal annealing, respectively (Fig. S10 and S7†). This result indicates that the chromophores of the co-assembly stem from Pt8. In addition, the PL emissions of the chiral co-assemblies are in agreement with those of Pt8 before and after thermal annealing (Fig. 3a–c and 1b–d), demonstrating that the chiral inducer *R*/*S*-M has little effect on the PL behaviors of chiral co-assemblies.

Notably, the 180 °C-annealed films (*R*-M)_0.03_-(Pt8)_0.97_ also exhibit mechanochromic PL behaviors, with an emission color change from green to orange-red (Fig. 3d). The reversible mechanochromism of the chiral co-assembly (*R*-M)_0.03_-(Pt8)_0.97_ under mechanical stimuli and thermal annealing at 180 °C was monitored at the emission maximum, demonstrating multiple cycles without perceivable performance degradation (Fig. 3e). The significant color and emission changes in response to mechanical grinding and annealing treatments on (*R*-M)_0.03_-(Pt8)_0.97_ enable rapid writing and erasure of “MCPL” through mechanochromism and thermal annealing processes. Writing could be done on the prepared film by the external mechanical force (Video S2†) and erasure by thermal annealing treatment at 180 °C (Video S3† and Fig. 3f). The pronounced mechanochromism of the chiral co-assemblies suggests their potential for optical anticounterfeiting. As depicted in Fig. 3g, solutions of Pt8 and (*R*/*S*-M)_0.03_-(Pt8)_0.97_ were utilized as security inks to paint three anticounterfeiting patterns of star, flower and heart, respectively. The created patterns exhibit distinct emission color changes from orange-red to green upon thermal annealing at 180 °C. When an external mechanical force was applied, the recovery of orange-red emissions was observed, confirming that the achiral Pt8 and chiral co-assemblies (*R*/*S*-M)_0.03_-(Pt8)_0.97_ hold great potential in optical anticounterfeiting.

### Color-switchable CPL of chiral co-assemblies upon thermal annealing and mechanical treatments

First, we measured the circular dichroism (CD) and CPL spectra of the chiral inducer *R*/*S*-M in spin-coated films. Good mirror-image Cotton peaks in the CD spectra were observed at 204 nm (|*g*_abs_| = 2.17 × 10^−3^), 239 nm (|*g*_abs_| = 2.41 × 10^−3^) and 326 nm (|*g*_abs_| = 1.40 × 10^−3^) for *R*/*S*-M (Fig. S11a†). However, very weak CPL signals were detected at 390 nm (|*g*_em_| = 1.45 × 10^−3^) for *R*/*S*-M (Fig. S11†), which is consistent with our previous report.^9^

**Fig. 4:**
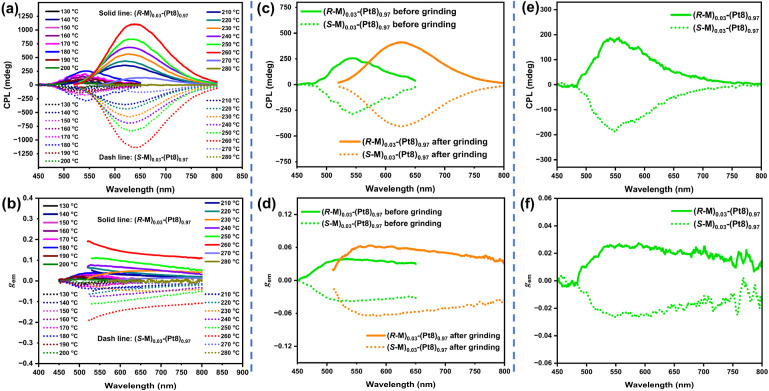
Temperature-responsive CPL spectra (a) and *g*_em_ values *vs.* wavelength (b) of (*R*/*S*-M)_0.03_-(Pt8)_0.97_; mechanically-responsive CPL spectra (c) and *g*_em_ values *vs.* wavelength (d) of 180 °C-annealed (*R*/*S*-M)_0.03_-(Pt8)_0.97_ before and after mechanical grinding; recovered CPL spectra (e) and *g*_em_ values *vs.* wavelength (f) of the grinding films (*R*/*S*-M)_0.03_-(Pt8)_0.97_ after reheating to 180 °C.

Then, the chiroptical properties of chiral co-assembly *R*/*S*-M-Pt8 were also investigated. At different molar ratios (1–10%) of *R*/*S*-M, the co-assembled films *R*/*S*-M-Pt8 exhibit weak CD bands centered at 293 nm and 350 nm (Fig. S12a†). Notably, (*R*/*S*-M)_0.03_-(Pt8)_0.97_ exhibits the best chirality induction ability from the chiral inducer *R*/*S*-M to the achiral emitter Pt8. After thermal annealing at 180 °C, (*R*/*S*-M)_0.03_-(Pt8)_0.97_ shows a new intense CD band in the range of 382–500 nm (*λ*_peak_ = 395 nm, |*g*_abs_| = 3.94 × 10^−3^, Fig. S12b†), originating from MLCT transitions of monomer Pt8. Increasing the temperature to 260 °C, a more intense and red-shifted CD band was located at 440 nm (|*g*_abs_| = 2.21 × 10^−2^, Fig. S12b†), which is consistent with the MMLCT transitions of the excimer in annealed films. This indicates that the LC matrix can greatly facilitate the chirality amplification effect and chirality induction ability.^18*a*^ Unannealed films of *R*/*S*-M-Pt8 show weak orange-red CPL at room temperature (*λ*_em_ = 625 nm, Fig. S13†), whereas the neat film of Pt8 is CPL-silent (Fig. S14†). The co-assembled films (*R*/*S*-M)_0.03_-(Pt8)_0.97_, with a molar ratio of 3% *R*/*S*-M, exhibit the highest CPL intensity and the largest |*g*_em_| value of 0.017 (Fig. S13†), indicating the best matched ratio between the chiral inducer and achiral homoleptic complex Pt8 in the co-assembly process. Therefore, (*R*/*S*-M)_0.03_-(Pt8)_0.97_ was selected for thermal annealing treatments to improve CPL activity. As expected, the chiral co-assembled film (*R*/*S*-M)_0.03_-(Pt8)_0.97_ exhibits more intense and temperature-responsive CPL behaviors after thermal annealing at different temperatures of 130–280 °C. Specifically, (*R*/*S*-M)_0.03_-(Pt8)_0.97_ emit green CPL signals at approximately 545 nm when annealed at 130–200 °C (Fig. 4a), with the largest |*g*_em_| value of 0.038 detected after annealing at 180 °C (Fig. 4b). Surprisingly, upon the temperature increasing to 210–280 °C, the CPL signals shift from green to orange-red and red regions. Among these annealed films, 260 °C-annealed films (*R*/*S*-M)_0.03_-(Pt8)_0.97_ display the most intense red CPL signals (*λ*_em_ = 640 nm, |*g*_em_| = 0.133) (Fig. 4a and b), stemming from MMLCT transitions and indicating that the excimer of Pt8 is in a chirality coupling environment at the LC state.^29^ The CPL intensity and |*g*_em_| values of orange-red or red CPL are significantly higher than those of green CPL, demonstrating that the chirality induction/transfer from *R*/*S*-M to the excimer of Pt8 is more effective than that to the monomer.

In CPL measurements, a brilliant orange-red facula emerged on the 180 °C-annealed chiral co-assembled films (*R*-M)_0.03_-(Pt8)_0.97_ when scraped with tweezers (Fig. S15†). This phenomenon has aroused our vigilance and attention. Given the mechanochromism behaviors observed in chiral co-assemblies, we aimed to investigate if these co-assemblies exhibit mechanically-responsive CPL when an external force applied. Here, chiral co-assemblies (*R*/*S*-M)_0.03_-(Pt8)_0.97_ were selected to probe mechanically-responsive CPL in the 180 °C-annealed films. As described earlier, 180 °C-annealed films (*R*/*S*-M)_0.03_-(Pt8)_0.97_ emit green CPL (*λ*_em_ = 545 nm, |*g*_em_| = 0.038) without mechanical force treatments (Fig. 4c and d). Surprisingly, when treated with mechanical grinding, the CPL emissions quickly shifted to orange-red (*λ*_em_ = 625 nm, |*g*_em_| = 0.058), with a significant increase in intensity (Fig. 4c and d), demonstrating the mechanically-responsive CPL of (*R*/*S*-M)_0.03_-(Pt8)_0.97_. Notably, the initial green CPL can be recovered *via* reheating the ground films (*R*/*S*-M)_0.03_-(Pt8)_0.97_ to 180 °C (Fig. 4e and f). These results indicated that the fast CPL emission color switching of transiently responsive (*R*/*S*-M)_0.03_-(Pt8)_0.97_ can be achieved *via* mechanical grinding.

### Mechanism of chiral co-assembly upon thermal annealing and mechanical stimuli

**Fig. 5:**
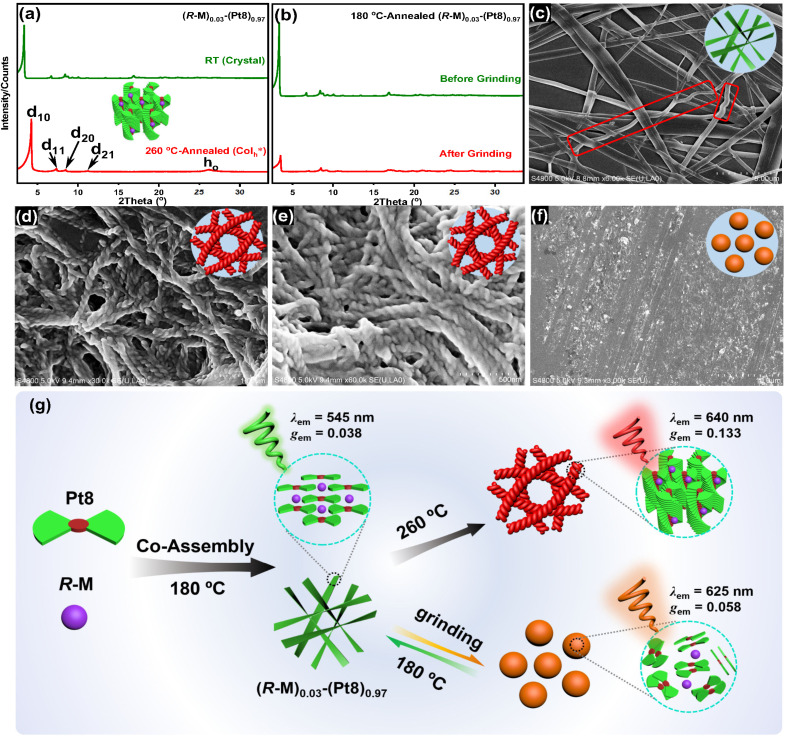
XRD patterns of the drop-cast film (*R*-M)_0.03_-(Pt8)_0.97_ before and after thermal annealing at 260 °C (a); XRD patterns of 180 °C-annealed (*R*-M)_0.03_-(Pt8)_0.97_ before and after mechanical grinding (b); SEM images of (*R*-M)_0.03_-(Pt8)_0.97_ annealed at 180 (c) and 260 °C (d); SEM image of (*S*-M)_0.03_-(Pt8)_0.97_ annealed at 260 °C (e); SEM image of 180 °C-annealed (*R*-M)_0.03_-(Pt8)_0.97_ after mechanical grinding (f); ((c) film, 1.0 × 10^−4^ mol L^−1^ in chloroform; (d–f) film, 1.0 × 10^−3^ mol L^−1^ in chloroform); schematic illustrations of the reversible CPL emission color switching of (*R*-M)_0.03_-(Pt8)_0.97_ upon thermal annealing and mechanical grinding (g).

Supramolecular chiral co-assembly is a significant protocol to endow an achiral emitter with chiroptical properties, and we have revealed that intermolecular π–π stacking interactions could effectively enhance the capability of chirality induction/transfer.^7*b*,9,22^ Compared to chiral liquid crystals with a cholesteric (N*-LCs), chiral smectic (SmC*/SmA*) and twist grain boundary (TGB*) mesophase, the chiral co-assembly of a columnar mesophase is less studied.^18*a*,30^ Scanning electron microscopy (SEM), POM and XRD measurements were carried out to probe the possible chiral co-assembly mechanism of (*R*/*S*-M)_0.03_-(Pt8)_0.97_ upon thermal annealing. No significant change in POM textures was observed between pure Pt8 (Fig. 2c) and the co-assemblies (*R*/*S*-M)_0.03_-(Pt8)_0.97_ (Fig. S16a and b†), indicating similar mesophases and good compatibility between the achiral homoleptic liquid crystal and the chiral inducer. SEM experiments of (*R*/*S*-M)_0.03_-(Pt8)_0.97_ were performed at 1.0 × 10^−3^ and 1.0 × 10^−4^ mol L^−1^ respectively in drop-cast films. As shown in Fig. S17a, b, S18a and b,† irregularly-distributed nanobelts with sharp fringes were clearly observed in the unannealed co-assembly (*R*/*S*-M)_0.03_-(Pt8)_0.97_. In contrast, these nano-superstructures transform into more twisted nanobelts after thermal annealing at 180 °C (Fig. 5c, S17c, d and S18c†), indicating that a quasi-chiral molecular arrangement of (*R*/*S*-M)_0.03_-(Pt8)_0.97_ is found to occur during the co-assembly process,^6*f*,31^ which may be a crucial factor for the weak green CPL signals in the 180 °C-annealed films (Fig. 4a and b). More intriguingly, when the temperature was increased to 260 °C, the twisted nanobelts of the chiral co-assembly gradually transformed into distinct nanofibers. Therein the left- and right-handed helices were respectively observed for (*R*-M)_0.03_-(Pt8)_0.97_ (Fig. 5d and S19a†) and (*S*-M)_0.03_-(Pt8)_0.97_ (Fig. 5e and S19b†), indicative of the highly ordered helical nanostructures. In addition, the XRD patterns of (*R*/*S*-M)_0.03_-(Pt8)_0.97_ at 260 °C demonstrated their helical Col_h_
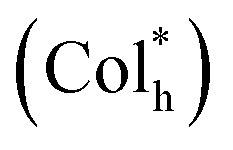
 mesophase, as reflected by the four typical reflections of *d*_10_, *d*_11_, *d*_20_ and *d*_21_ in the small-angle region (Fig. 5a and S16c†). Notably, a broad diffuse peak (*h*_0_) of *d* = 3.41 Å was observed at 2*θ* = 26.19°, attributed to the intermolecular π–π stacking interactions along the column. Combining SEM, POM and XRD analyses, we deduced that (*R*/*S*-M)_0.03_-(Pt8)_0.97_ forms the 
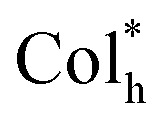
 mesophase *via* intermolecular π–π stacking interactions, facilitating the MMLCT transitions of Pt8 in the helical nanofibers. This result promotes the generation and amplification of CPL signals in the chiral co-assembly process. In the dynamic reversible mechanically-responsive process (Fig. 5g), the ordered molecular stacking of 180 °C-annealed co-assemblies in the twisted nanobelts (Fig. 5b, c, S16d and S18c†) was disrupted and converted into irregularly-distributed nanoparticles (Fig. 5b, f, S16d, and S19c†). The external mechanical force promoted the formation of Pt8 excimers in nanoparticles, resulting in a more efficient MMLCT chirality coupling environment.^29^ Reheating the ground film (*R*/*S*-M)_0.03_-(Pt8)_0.97_ to 180 °C restructured the twisted nanobelts (Fig. S20†), thereby leading to the restoration of the ordered molecular arrangement and the initial green CPL (Fig. 4e and f).

### Mechanically-modulated information encryption

**Fig. 6:**
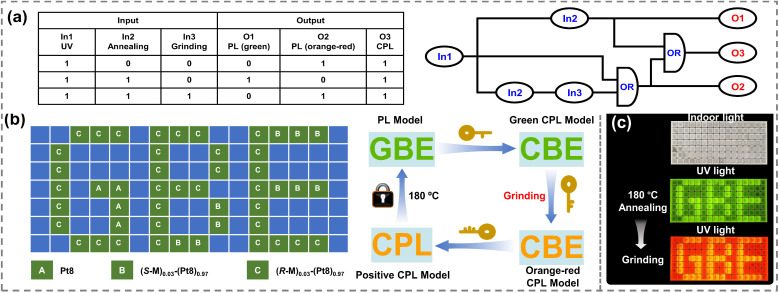
(a) The truth table and schematic diagrams of a logic gate based on (*R*-M)_0.03_-(Pt8)_0.97_ (symbolic representation of the integrated logic gate system using 365 nm excitation, annealing (at 180 °C) and grinding as inputs 1, 2 and 3, respectively. Green PL, orange-red PL and the CPL signal were defined as O1, O2 and O3); (b) the schematic diagrams of the recyclable mechanically-modulated information encryption and decryption system; (c) the real images of information encryption devices after 180 °C and grinding.

Based on the dynamic reversible CPL emission color switching of chiral co-assemblies, a chiroptical integrated logic system with multiple information outputs was designed. The corresponding truth table and schematic diagram are depicted in Fig. 6a. In order to realize the goal of effectively constructing logic gates, the following conditions were defined: the unannealed film of the chiral co-assembly (*R*-M)_0.03_-(Pt8)_0.97_ was set as the initial state of the logical circuit. The external stimuli included UV light (365 nm, In1), thermal annealing (180 °C, In2) and grinding treatment (In3), with logic inputs “0” and “1” representing the absence and presence of the external stimulus, respectively. Green PL emission, orange-red PL emission, and CPL activity were designated as O1, O2, and O3. The outputs “0” and “1” indicated the absence and presence of the signals, respectively. Consequently, the logic gate could operate under different inputs and output the corresponding information based on the stimuli-responsive traces. These multi-channel input and output logical circuits can manage the development of higher security-level information encryption models. As illustrated in Fig. 6b and c, the initial films of Pt8, (*R*-M)_0.03_-(Pt8)_0.97_ and (*S*-M)_0.03_-(Pt8)_0.97_ were prepared upon thermal annealing at 180 °C and placed into a 16 × 7 pixel grid following a predefined pattern. Upon UV illumination (*λ* = 365 nm), the message containing the green letters “GBE” could be clearly observed with the naked eye. However, when the CPL tools were applied to analyze the array, the incorrect message “CBE” with green CPL emissions was disclosed. Subsequently, the orange-red CPL message “CBE” with anticounterfeiting functionality could be further verified by mechanical grinding, but the letters still represented false information. Finally, the true information “CPL” could only be identified through a positive CPL model. It is worth noting that the information revealed during the decryption process will be concealed again *via* thermal annealing treatments (180 °C), achieving recyclable encryption and decryption models. Such a mechanically-modulated information encryption model offers higher security and complexity compared to conventional encryption models.

## Conclusions

In summary, a transiently responsive mechanochromic liquid crystal, Pt8, was designed and constructed, enabling fast CPL emission color switching in chiral supramolecular co-assemblies. Combining it with the anchored chiral binaphthyl inducers *R*/*S*-M forms chiral co-assemblies (*R*/*S*-M)_0.03_-(Pt8)_0.97_, which assemble into twisted nanobelts (180 °C) and helical nanofibers (260 °C), exhibiting green (*λ*_em_ = 545 nm, *g*_em_ = 0.038) and red CPL (*λ*_em_ = 640 nm, *g*_em_ = 0.133), respectively. More importantly, upon mechanical grinding, the twisted nanobelts of 180 °C-annealed (*R*/*S*-M)_0.03_-(Pt8)_0.97_ instantaneously transform into nanoparticles, resulting in a fast switching of CPL emission color from green to orange-red (*λ*_em_: 545 → 625 nm, *g*_em_: 0.038 → 0.058). The initial green CPL of the nanobelts can be restored after reheating the ground films (*R*/*S*-M)_0.03_-(Pt8)_0.97_ to 180 °C. Based on the fast emission color switching of CPL, we demonstrate the applications of these transiently responsive co-assemblies for fast writing and erasure applications, and mechanically-modulated information encryption.

## Author contributions

G. Z., Q. H. L., Z. H. J. and W. T. G. carried out most of the investigation and methodology. G. Z. and Q. H. L. were in charge of writing – original draft with assistance from all authors. Y. X. C. supervised the work and writing – review & editing, project administration and funding acquisition.

## Conflicts of interest

There are no conflicts to declare.

## Supplementary Material

SC-016-D5SC02285A-s001

SC-016-D5SC02285A-s002

SC-016-D5SC02285A-s003

SC-016-D5SC02285A-s004

## Data Availability

The data that support the findings of this study are available within the article and the ESI.†
