# Supplementary material for: Fast emission color switching of circularly polarized luminescence in platinum(ii) liquid crystalline co-assembly
Source: Chem Sci. 2025 May 13;16(24):11049–57. doi: 10.1039/d5sc02285a (PMC12093386; doi:10.1039/d5sc02285a)
Supplement: SC-016-D5SC02285A-s001 [file SC-016-D5SC02285A-s001.pdf]

Supplementary Information (SI) for Chemical Science.
This journal is © The Royal Society of Chemistry 2025

Electronic Supplementary Information

Fast Emission Color Switching of Circularly Polarized Luminescence in Platinum(II) Liquid Crystalline Co-Assembly

*Guo Zou, Qihuan Li, Zhenhao Jiang, Wentong Gao and Yixiang Cheng**

Contents

1. Experimental Section S2
2. Synthetic Procedures and Characterization S3
3. Additional Table for ESI S8
4. Additional Figures for ESI S11
5. References S19

1. Experimental Section

Materials and Methods. All precursor reagents, solvents and high purity nuclear magnetic resonance (NMR) solvents were purchased from commercial sources (Sigma Aldrich, Adamas, TCI and Acros) and used as supplied unless otherwise indicated. Solvents were distilled from sodium/benzophenone (toluene, tetrahydrofuran, *n*-hexane) or calcium hydride (DCM, chloroform) under argon prior to use. The free N^N-cyclometalated ligand **A-7** (**Scheme S1**) was prepared *via* the serial reactions of Sonogashira,[1] Suzuki-Miyaura carbon–carbon coupling,[2] and the Sharpless copper-catalyzed Huisgen's 1,3-dipolar cycloaddition procedures.[3] All oxygen or moisture sensitive reactions were performed under argon atmosphere using standard Schlenk method. The ^{1}H NMR and ^{13}C NMR spectra were detected on Bruker avance III 400/500 MHz spectrometer. Chemical shifts were recorded as parts per million (ppm, δ) relative to tetramethylsilane (δ 0.00) and $CDCl_3$ (δ = 7.26, singlet). ^{1}H NMR splitting patterns are designated as singlet (s), doublet (d), triplet (t), multiplets (m) and *etc*. Mass spectrometry (MS) analyses were recorded on a Bruker Daltonics Ultrafle Xtreme MALDI-TOF MS, and α-cyano-4-hydroxycinnamic acid (α-CHCA) was used as supporting matrix in the MALDI-TOF MS measurements. Thermo-gravimetry analysis (TGA) was performed on a NETZSCH STA 449 ***F3*** Jupiter instrument in an atmosphere of N_2 at a heating rate of 10 ºC/min. The liquid crystalline textures were investigated and photographed using liquid crystal cells with a polarized optical microscope (POM) equipped with a Leitz-350 heating stage and an associated Nikon (D3100) digital camera. Temperature-dependent, powder X-ray diffraction was measured on a small & wide angle X-Ray scattering system (SAXSess mc^2, Cu-Kα, λ = 0.15418 nm). Scanning electron microscope (SEM) images were taken in Hitachi S-4800 field emission scanning electron microscopy. Differential scanning calorimetry (DSC) experiments were performed on a METTLER DSC 823e differential canning calorimeter at a scan rate of 10 ºC/min under the nitrogen atmosphere. Ultraviolet−visible (UV−*vis*) absorption spectra were measured at room temperature on a Shimadzu UV-3600 spectrophotometer. The photoluminescence (PL) emission spectra were measured on an Edinburgh FLS 980 instrument. Absolute quantum yield measured using the calibrated integrating sphere system at room temperature. The time-resolved measurements were taken on an Edinburgh FLS 980 instrument to measure the excited state lifetime. A diode laser with λ = 365 nm was used as the excitation source, and the time-correlated single photo count (TCSPC) method was used to collect photos.

Computational Details. Computational studies were carried out using Gaussian 16 program package.[4] For density functional theory (DFT) and time-dependent DFT (TDDFT) calculations, B3LYP[5] hybrid density function and 6-311g(d) basis was used for nonmetal atoms along with SMD solvent model (solvent: chloroform).[6] For platinum, a Stuttgart−Dresden (SDD) effective core potential was employed. S_1 to S_{60} excited states were calculated based on the optimized structure in its ground state from DFT results, and the UV−*vis* spectrum was simulated using Multiwfn software.[7] The

empirical dispersion correction D3 with Becke-Johnson damping (GD3BJ) was used for all calculations.[8-12]

Thermal Annealing Treatments. The thin films were all prepared by dissolving the corresponding sample in chloroform (6 mg mL^{-1}), followed by spin coating (1000 rpm, 30 s) onto the quartz plate (1 cm × 3 cm). The above quartz plates were put on the hot stage at established temperature and the films were kept at this temperature for 15 min. Finally, the quartz plates were moved to room temperature.

2. Synthetic Procedures and Characterization

2.1 Synthesis of Achiral Homoleptic Pt(II) Complex Pt8

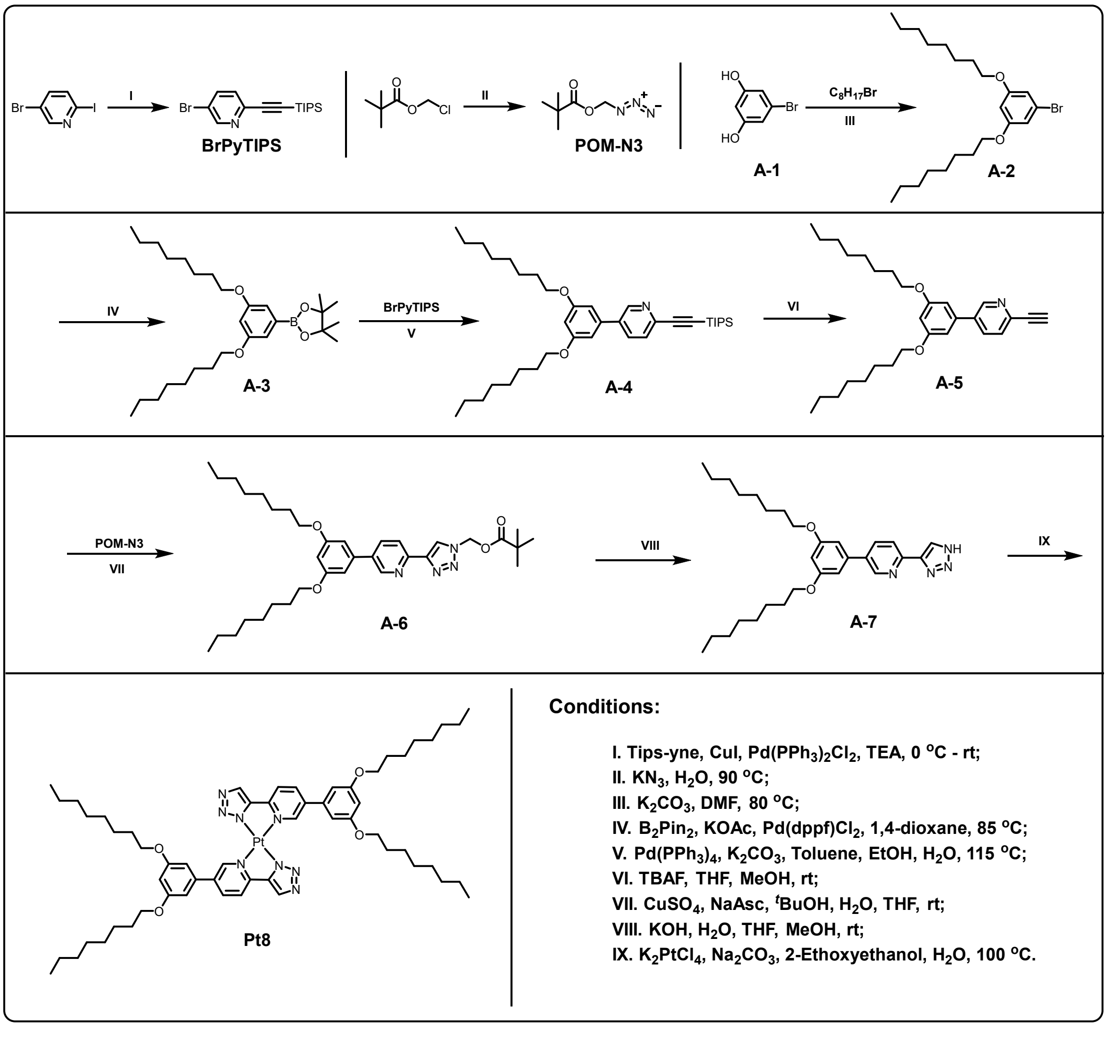

Scheme S1. Synthetic route of homoleptic Pt(II) complex **Pt8**.

Synthesis of compound BrPyTIPS

To a mixture of 5-bromo-2-iodopyridine (5.00 g, 17.61 mmol), ethynyltriisopropyl-silane (3.24 g, 17.79 mmol), $Pd(PPh_3)_2Cl_2$ (618.10 mg, 880.61 μmol) and CuI (167.71 mg, 880.61 μmol) in triethylamine (TEA, 50 mL) and acetonitrile (MeCN, 50 mL). Under the protection of argon, the mixture was stirred at RT overnight. Then the solvent was removed *via* evaporating and the residue dissolved in DCM (100 mL), then washed with water (3 × 50 mL), dried over anhydrous Na_2SO_4 and filtrated. The filtrate was evaporated to remove the solvent and the residue was passed through a flash silica gel column using *n*-hexane as the eluent to give **BrPyTIPS** as a clear colorless oil (5.00 g, 84%). ^{1}H NMR (400 MHz, $CDCl_3$) δ 8.64 (d, J = 1.9 Hz, 1H), 7.77 (dd, J = 8.3, 2.4 Hz, 1H), 7.35 (dd, J = 8.3, 0.4 Hz, 1H), 1.16 – 1.12 (m, 21H).

Synthesis of compound POM-N3

To a solution of KN_3 (2.96 g, 36.52 mmol) in deionized water (80 mL) was added chloromethyl pivalate (5.00 g, 33.20 mmol). The mixture was stirred at 90 ºC overnight under the inert gas atmosphere. The resultant mixture was extracted with ethyl acetate (EA, 80 mL) for three times. The final combined organic layer was dried with anhydrous Na_2SO_4 and filtered. The solvent was removed by evaporating to get **POM-N3** as a colorless liquid (4.90 g, 94%). ^{1}H NMR (400 MHz, $CDCl_3$) δ 5.14 (s, 2H), 1.25 (s, 9H). The NMR matched the reported previously.[13]

Synthesis of compound A-2[14-17]

To a mixture of 5-bromobenzene-1,3-diol (**A-1**, 3.00 g, 15.87 mmol), 1-bromooctane (7.66 g, 39.68 mmol) and potassium carbonate (8.77 g, 63.49 mmol) was added a degassed mixture of N, N-Dimethylformamide (DMF, 60 mL). The mixture was reacted at 80 ºC for 48 h under the protection of argon. After cooled to RT, the mixture was poured into water (100 mL) and extracted with DCM (3 × 50 mL). The combined organic layer was dried over anhydrous Na_2SO_4 and filtered. The filtrate was evaporated to remove the solvent and the residue was passed through a flash silica gel column using PE/DCM (10:1) as the eluent to get **A-2** as a colorless oil (6.30 g, 96%). ^{1}H NMR (400 MHz, $CDCl_3$) δ 6.63 (d, J = 2.2 Hz, 2H), 6.36 (t, J = 2.2 Hz, 1H), 3.90 (t, J = 6.6 Hz, 4H), 1.80 – 1.70 (m, 4H), 1.47 – 1.38 (m, 4H), 1.36 – 1.24 (m, 16H), 0.89 (dd, J = 9.2, 4.6 Hz, 6H).

Synthesis of compound A-3

A mixture of **A-2** (3.00 g, 7.26 mmol), bis(pinacolato)diboron (2.21 g, 8.71 mmol), potassium acetate (2.14 g, 21.77 mmol), [1,1'-bis(dipheny- lphosphino)-ferrocene] dichloropalla- dium complex with dichloromethane (1:1) (592.57 mg, 725.62 μmol), and 1,4-dioxane (60 mL) was stirred at 85 ºC for 12 h under argon atmosphere. The resulting mixture was cooled to RT, poured into ice-water (100 mL) and then extracted with DCM (3 × 50 mL). The combined organic layers were washed with water and dried over anhydrous Na_2SO_4 and filtrated. The filtrate was evaporated to remove the solvent and the residue was passed through a flash silica gel column using PE/EA (10:1) as the eluent to give **A-3** as a brown oil (3.30 g, 99%). ^{1}H NMR (400 MHz, $CDCl_3$) δ 6.92 (d, J = 2.4 Hz, 2H), 6.55 (t, J = 2.4 Hz, 1H), 3.96 (t, J = 6.5 Hz, 4H), 1.79 – 1.70 (m, 4H), 1.47 – 1.39 (m, 4H), 1.33 (s, 12H), 1.33 – 1.24 (m, 16H), 0.89 (t, J = 6.9 Hz, 6H).

Synthesis of compound A-4

To a mixture of **A-3** (3.00 g, 6.51 mmol), **BrPyTIPS** (2.42 g, 7.17 mmol), potassium carbonate (2.70 g, 19.54 mmol) and tetrakis(triphenylphosphine) palladium ($Pd(PPh_3)_4$, 752.82 mg, 651.46 μmol) was added a degassed mixture of toluene (40 mL), water (20 mL) and ethanol (20 mL). The mixture was refluxed (115 ºC) for 24 h under the protection of argon. After cooled to RT, the mixture was poured into water (100 mL) and extracted with DCM (3 × 30mL). The combined organic layer was dried over anhydrous Na_2SO_4 and filtered. The filtrate was evaporated to remove the solvent and the residue was passed through a flash silica gel column using PE/EA (30:1) as the eluent to give **A-4** as a brown oil (3.00 g, 78%). ^{1}H NMR (400 MHz, $CDCl_3$) δ 8.80 (d, J = 1.6 Hz, 1H), 7.83 (dd, J = 8.0, 1.5 Hz, 1H), 7.53 (d, J = 8.1 Hz, 1H), 6.66 (d, J = 2.1 Hz, 2H), 6.50 (t, J = 2.0 Hz, 1H), 3.99 (t, J = 6.5 Hz, 4H), 1.84 – 1.75 (m, 4H), 1.46 (dd, J = 15.0, 7.0 Hz, 4H), 1.39 – 1.24 (m, 16H), 1.21 – 1.12 (m, 21H), 0.89 (t, J = 6.8 Hz, 6H).

Synthesis of compound A-5

To a solution of **A-4** (2.77 g, 4.68 mmol) and tetrabutylammonium fluoride (TBAF, 1.84 g, 7.02mmol) in tetrahydrofuran (THF, 20 mL) and methanol (MeOH; 1 mL). The mixture was stirred at RT for 5 min under the air atmosphere. After reaction completion (detected by thin layer chromatography, TLC), the mixture was evaporated to remove the solvent and the residue was passed through a flash silica gel column using PE/EA (10:1) as the eluent to give **A-5** as a colorless oil (2.00 g, 98%). ^{1}H NMR (400 MHz, $CDCl_3$) δ 8.81 (d, J = 1.7 Hz, 1H), 7.88 (dd, J = 8.1, 2.3 Hz, 1H), 7.56 (d, J = 8.1 Hz,

1H), 6.68 (d, $J = 2.2$ Hz, 2H), 6.51 (t, $J = 2.2$ Hz, 1H), 3.99 (t, $J = 6.6$ Hz, 4H), 3.26 (s, 1H), 1.85 – 1.75 (m, 4H), 1.51 – 1.42 (m, 4H), 1.38 – 1.26 (m, 16H), 0.89 (t, $J = 6.8$ Hz, 6H).

Synthesis of compound A-6

A solution of **A-5** (2.00 g, 4.59 mmol) and **POM-N3** (1.08 g, 6.89 mmol) in THF (30 mL), was added copper sulfate pentahydrate (82.47 mg, 459.08 μmol) and sodium ascorbate (300.12 mg, 1.51 mmol) in deionized water (10 mL) and tert-butanol (5 mL). The mixture was stirred overnight at RT in air. After reaction completion (detected by TLC), the mixture was extracted with EA (3 × 20 mL). The combined organic layer was washed with water (3 × 50 mL), and dried over anhydrous Na_2SO_4 and filtrated. The filtrate was evaporated to remove the solvent and the residue was passed through a flash silica gel column using PE/EA (5:1) as the eluent to give **A-6** as a light brown oil (2.50 g, 91 %). ^{1}H NMR (400 MHz, $CDCl_3$) δ 8.82 (d, $J = 2.0$ Hz, 1H), 8.48 (s, 1H), 8.25 (d, $J = 8.2$ Hz, 1H), 7.99 (d, $J = 7.1$ Hz, 1H), 6.72 (d, $J = 2.1$ Hz, 2H), 6.51 (t, $J = 2.1$ Hz, 1H), 6.32 (s, 2H), 4.00 (t, $J = 6.6$ Hz, 4H), 1.85 – 1.76 (m, 4H), 1.52 – 1.43 (m, 4H), 1.38 – 1.27 (m, 16H), 1.21 (s, 9H), 0.89 (t, $J = 6.8$ Hz, 6H).

Synthesis of compound A-7[3]

A solution of **A-6** (1.00 g, 1.69 mmol) in THF (30 mL), was added KOH (141.96 mg, 2.53 mmol; 2 M in water) and MeOH (3 mL). The mixture was stirred at RT in ambient condition for 30 min, after reaction completion (detected by TLC), the mixture was extracted with EA (3 × 20 mL). The combined organic layer was washed with water (3 × 50 mL), and dried over anhydrous Na_2SO_4 and filtrated. The filtrate was evaporated to remove the solvent to give crude product **A-7** as a colorless oil (800.00 mg, 99%). This product was directly used to next step without further purification.

Synthesis of homoleptic Pt(II) complex Pt8[13, 18]

To a mixture of **A-7** (500.00 mg, 1.04 mmol), K_2PtCl_4 (173.43 mg, 417.81 μmol) and Na_2CO_3 (442.83 mg, 4.18 mmol) in 2-Ethoxyethanol (10 mL) and water (3.5 mL), was stirred at 100 °C for 24 h. After the mixture was cooled to RT, it was extracted with DCM (3 × 10 mL). The combined organic layer was washed with water (3 × 20 mL), and dried over anhydrous Na_2SO_4 and filtrated. The filtrate was evaporated to remove the solvent and the residue was passed through a flash silica gel column using DCM/EA (10:1) as the eluent to give **Pt8** as a white solid (453.00 mg, 94%). ^{1}H NMR (400 MHz, $CDCl_3$) δ 10.14 (s, 2H), 7.75 (dd, $J = 8.2$, 1.3 Hz, 2H), 7.49 (s, 2H), 7.15 (d, $J = 8.2$ Hz, 2H), 6.55 (d, $J = 1.5$ Hz, 4H), 6.38 (s, 2H), 3.94 (t, $J = 6.5$ Hz, 8H), 1.86 – 1.77 (m,

8H), 1.48 (d, J = 6.9 Hz, 8H), 1.43 – 1.31 (m, 32H), 0.93 (t, J = 6.8 Hz, 12H). ^{13}C NMR (126 MHz, $CDCl_3$) δ 160.55, 150.33, 149.38, 143.79, 135.65, 135.29, 134.03, 130.75, 118.81, 104.08, 102.01, 68.21, 31.96, 29.61, 29.46, 29.42, 26.25, 22.75, 14.17. MALDI-TOF MS m/z (α-CHCA as matrix) calcd. for $C_{58}H_{83}N_8O_4Pt^+$ $[M+H]^+$: m/z = 1150.619, found: m/z = 1150.933.

2.2 Synthesis of Chiral Inducer *R*/*S*-M[19]

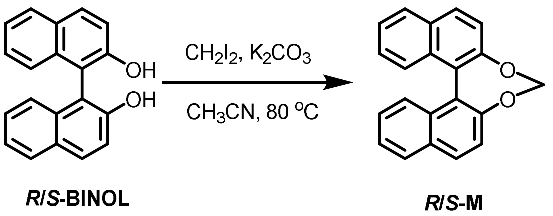

Scheme S2. Synthetic route of chiral inducer ***R*/*S*-M**.

To a mixture of ***R*-BINOL** (1.00 g, 3.49 mmol), CH_2I_2 (1.87 g, 6.98 mmol) and anhydrous K_2CO_3 (1.93 g, 13.97 mmol) was added CH_3CN (30 mL) under stirring magnetically, and the mixture was refluxed at 80 ºC for 12 h. After cooled to room temperature, the reaction mixture was filtered and concentrated under a reduced pressure. The crude product was purified by a flash silica gel column using DCM as the eluent to afford ***R*-M** as a white solid (1.02 g, 98%). ^1H NMR (400 MHz, $CDCl_3$) δ 7.94 (dd, J = 17.6, 8.8 Hz, 4H), 7.49 (d, J = 16.0, 8.4 Hz, 4H), 7.45-7.41 (m, 2H), 7.30-7.26 (m, 2H), 5.68 (s, 2H).

***S*-M** (990.00 mg, yield = 95 %) was synthesized as a white solid, according to the same method of ***R*-M**.

3. Additional Table for ESI

Table S1. Computed excitation energies and oscillator strengths for the $S_1 \rightarrow S_{60}$ transitions of homoleptic Pt(II) complex **Pt8**.

S_n	*E* (eV)	*λ* (nm)	*f*	transitions
S_1	3.1833	389.48	0.0109	H → L 93.6%, H-1 → L+1 5.0%
S_2	3.1835	389.46	0.0027	H-1 → L 93.6%, H → L+1 5.0%
S_3	3.3495	370.16	0.2714	H-2 → L 78.8%, H-4 → L 18.2%
S_4	3.4859	355.67	0	H-3 → L 96.1%
S_5	3.6575	338.99	0.0049	H → L+1 93.2%, H-1 → L 5.1%
S_6	3.6576	338.98	0.008	H-1 → L+1 92.2%, H → L 5.0%
S_7	3.7157	333.68	0.1948	H-4 → L 77.4%, H-2 → L 18.6%
S_8	3.8319	323.56	0	H-2 → L+1 83.1%, H-4 → L+1 10.1%
S_9	3.8613	321.09	0.0063	H-7 → L 95.6%
S_{10}	3.9387	314.78	0.5358	H-3 → L+1 69.3%, H → L+2 13.8%, H-1 → L+3 8.2%
S_{11}	3.9477	314.07	0.0077	H-1 → L+2 60.6%, H → L+3 36.0%
S_{12}	3.9495	313.92	0.3195	H → L+2 47.2%, H-1 → L+3 27.9%, H-3 → L+1 21.0%
S_{13}	4.1002	302.39	0.0788	H-5 → L 46.8%, H-8 → L 33.0%, H-3 → L+1 5.2%
S_{14}	4.1095	301.7	0.032	H-4 → L+4 47.4%, H-2 → L+4 27.0%, H-5 → L 6.9%
S_{15}	4.1134	301.42	0.0001	H-4 → L+1 71.4%, H-2 → L+1 11.5%, H-6 → L 10.4%
S_{16}	4.1966	295.44	0.0001	H-6 → L 70.5%, H-4 → L+1 13.3%
S_{17}	4.2166	294.04	0.0971	H-2 → L+2 41.7%, H-5 → L 18.1%, H-8 → L 17.6%, H-3 → L+3 10.8%
S_{18}	4.2561	291.31	0.0013	H-7 → L+1 89.7%
S_{19}	4.2766	289.91	0	H-7 → L+4 58.6%, H-3 → L+2 12.9%, H-2 → L+3 10.6%, H-8 → L+1 6.3%
S_{20}	4.2866	289.24	0	H → L+3 60.3%, H-1 → L+2 36.4%
S_{21}	4.2868	289.22	0.0019	H-1 → L+3 61.1%, H → L+2 36.7%
S_{22}	4.3033	288.11	0.0003	H-2 → L+3 33.6%, H-3 → L+2 26.9%, H-7 → L+4 20.5%, H-6 → L 8.7%
S_{23}	4.338	285.81	1.0144	H-8 → L 40.8%, H-2 → L+2 30.8%, H-5 → L 18.8%
S_{24}	4.3917	282.31	0.0004	H-9 → L 94.1%
S_{25}	4.4358	279.51	0	H-3 → L+2 49.3%, H-2 → L+3 39.6%, H-4 → L+3 5.9%

S_{26}	4.4687	277.45	0.0686	H-3 → L+3 71.2%, H-4 → L+2 12.6%, H-2 → L+2 11.0%
S_{27}	4.491	276.07	0.0009	H-10 → L 65.2%, H-2 → L+4 9.6%, H-9 → L+1 6.0%, H-4 → L+4 5.3%, H-8 → L+4 5.0%
S_{28}	4.5114	274.82	0.0028	H-10 → L 23.8%, H-2 → L+4 20.6%, H-8 → L+4 15.7%, H-4 → L +4 14.6%, H-4 → L+2 11.3%, H-5 → L+4 6.3%
S_{29}	4.5621	271.77	0.0002	H-1 → L+4 88.6%, H-3 → L+4 9.3%
S_{30}	4.5652	271.59	0.0013	H → L+4 98.1%
S_{31}	4.6028	269.37	0.0334	H-4 → L+2 65.7%, H-4 → L+4 9.2%, H-3 → L+3 7.8%
S_{32}	4.6048	269.25	0	H-3 → L+4 72.1%, H-4 → L+3 11.7%, H-1 → L+4 10.3%
S_{33}	4.6144	268.69	0	H-5 → L+1 76.0%, H-4 → L+3 8.5%, H-8 → L+1 5.7%
S_{34}	4.6364	267.41	0	H-4 → L+3 67.9%, H-5 → L+1 12.2%, H-3 → L+4 8.9%
S_{35}	4.6573	266.21	0.1214	H-6 → L+1 88.4%
S_{36}	4.7713	259.85	0.0002	H-8 → L+1 72.7%, H-2 → L+3 6.7%, H-5 → L+1 5.3%
S_{37}	4.8095	257.79	0.0023	H-8 → L+4 48.7%, H-2 → L+4 27.0%, H-7 → L+3 7.9%
S_{38}	4.8211	257.17	0	H-7 → L+2 91.3%
S_{39}	4.8614	255.04	0.0002	H-7 → L+3 85.3%, H-8 → L+4 5.4%
S_{40}	4.9026	252.89	0.0006	H-9 → L+1 84.5%, H-10 → L 8.4%
S_{41}	4.9637	249.78	0.0587	H-5 → L+2 63.4%, H-6 → L+3 24.8%
S_{42}	4.9658	249.68	0	H-6 → L+2 40.4%, H-11 → L 27.1%, H-5 → L+3 22.1%
S_{43}	4.9939	248.27	0	H-10 → L+1 91.7%
S_{44}	5.0343	246.28	0.0005	H-11 → L 58.3%, H-5 → L+3 12.3%, H-6 → L+2 8.8%, H-13 → L 7.7%
S_{45}	5.1019	243.02	0.0345	H → L+5 47.4%, H-1 → L+6 21.9%, H-3 → L+8 7.4%, H-2 → L+7 5.2%
S_{46}	5.1045	242.89	0.0121	H-1 → L+5 45.8%, H → L+6 21.2%, H-2 → L+8 7.3%, H-3 → L+7 5.4%
S_{47}	5.1496	240.76	0.0599	H-8 → L+2 52.9%, H-12 → L 22.8%, H-6 → L+3 9.3%
S_{48}	5.1781	239.44	0	H-19 → L+4 44.4%, H-9 → L+4 43.3%
S_{49}	5.1933	238.74	0.0002	H-5 → L+3 48.8%, H-6 → L+2 30.9%, H-8 → L+3 10.4%

S_{50}	5.2258	237.25	0.0141	H-6 → L+3 57.4%, H-5 → L+2 22.3%, H-12 → L 8.1%, H-8 → L+2 5.8%
S_{51}	5.2704	235.25	0.1012	H-12 → L 59.7%, H-8 → L+2 28.9%
S_{52}	5.2927	234.26	0	H-8 → L+3 62.2%, H-13 → L 17.4%, H-5 → L+3 6.3%, H-6 → L+2 5.4%
S_{53}	5.3466	231.89	0	H-13 → L 62.6%, H-8 → L+3 17.1%, H-11 → L 7.4%
S_{54}	5.3538	231.58	0.0031	H-18 → L 93.1%
S_{55}	5.3847	230.25	0	H-9 → L+2 76.0%, H-19 → L 14.3%
S_{56}	5.4194	228.78	0.0003	H-19 → L 77.9%, H-9 → L+2 15.2%
S_{57}	5.4423	227.82	0.0041	H-5 → L+4 80.4%, H-8 → L+4 6.6%
S_{58}	5.4482	227.57	0	H-6 → L+4 86.5%, H-3 → L+4 5.0%
S_{59}	5.4533	227.36	0.029	H-11 → L+1 86.2%, H-13 → L+1 5.6%
S_{60}	5.4656	226.84	0.0003	H-9 → L+3 77.4%, H-10 → L+2 17.3%

4. Additional Figures for ESI

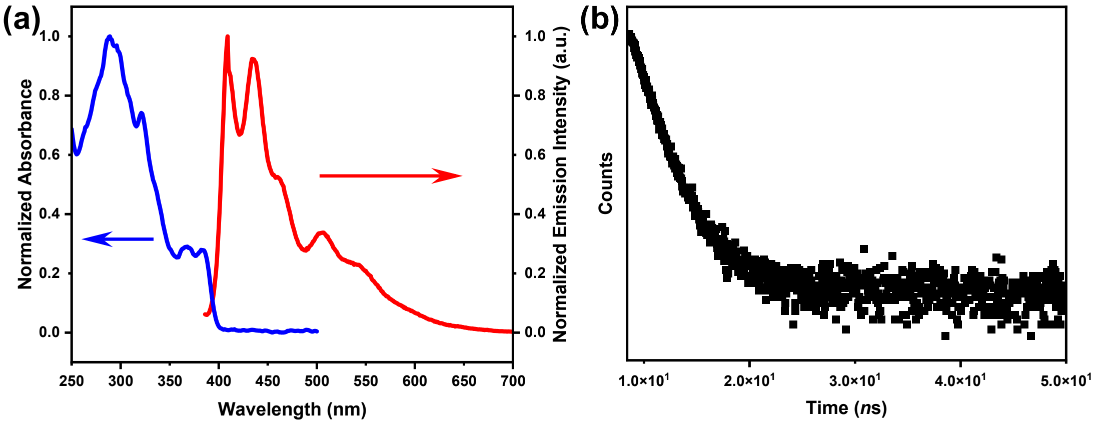

Fig. S1 UV−*vis* absorption and PL spectra (a), and transient PL decay (b) of homoleptic Pt(II) complex **Pt8** in chloroform (M = 1.0 × 10^{-6} mol L^{-1}).

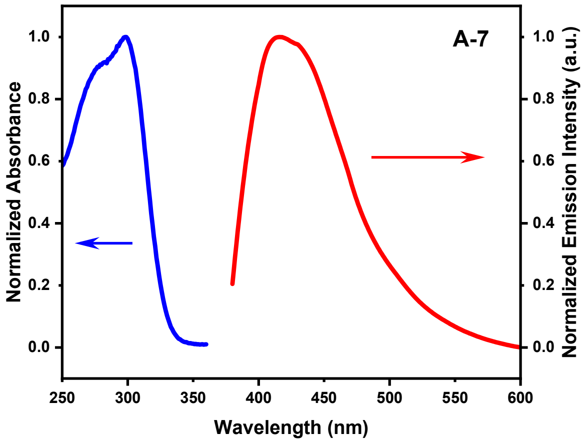

Fig. S2 UV−*vis* absorption and PL spectra of ligand **A-7** in chloroform (M = 1.0 × 10^{-6} mol L^{-1}).

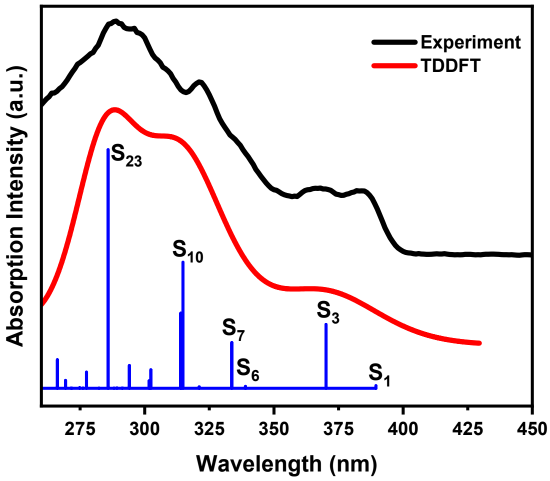

Fig. S3 TDDFT-predicated vertical excitations of **Pt8** (the experimental absorption spectrum is shown for comparison).

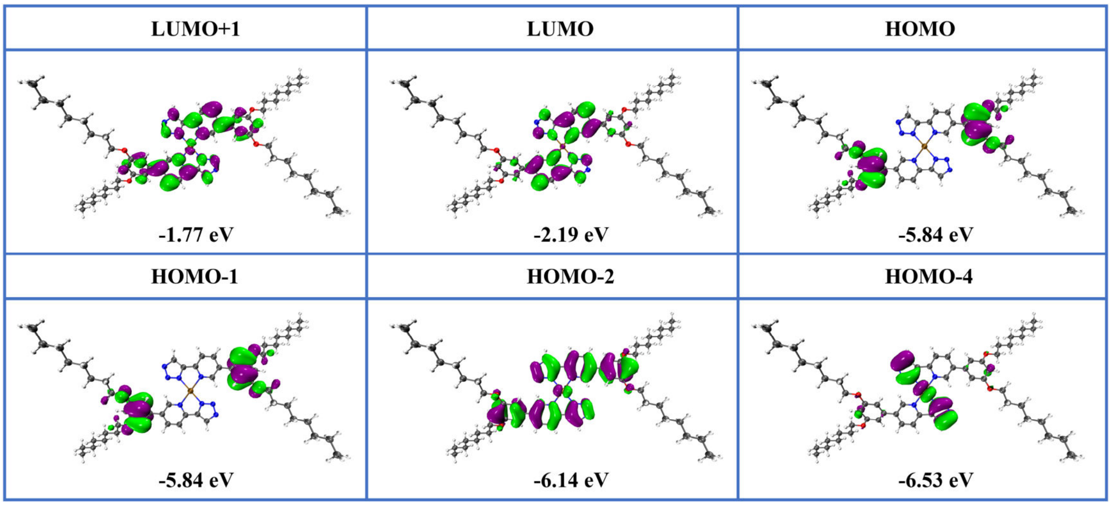

Fig. S4 Frontier molecular orbitals and energy level of **Pt8**.

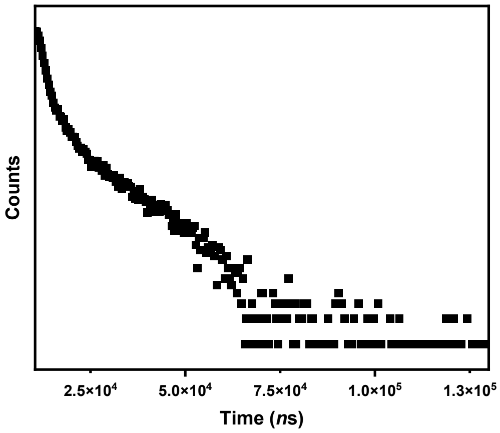

Fig. S5 Transient PL decay of homoleptic Pt(II) complex **Pt8** on spin-coated film.

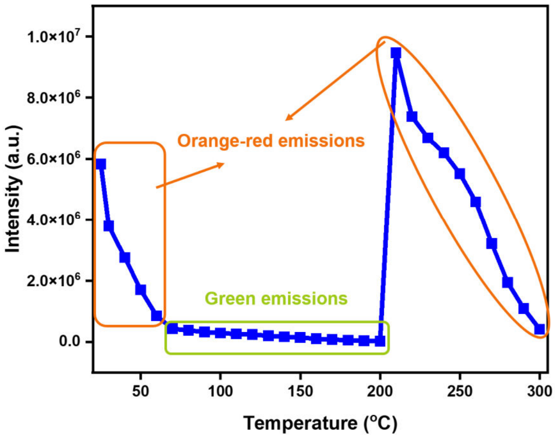

Fig. S6 The PL intensity (at λ_{peak}) *vs* the temperatures for **Pt8** on spin-coated film.

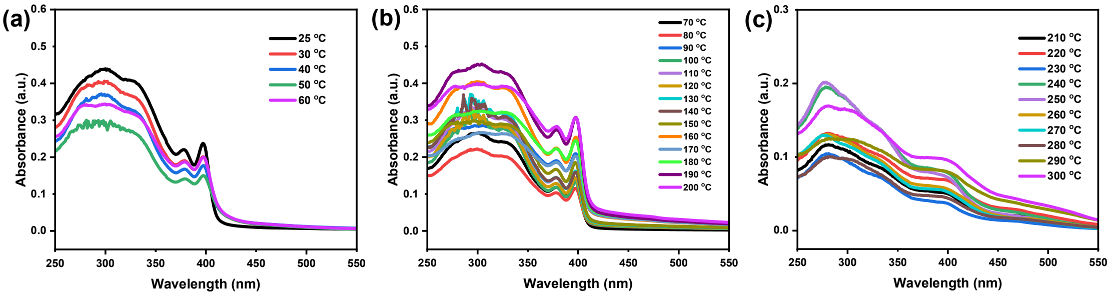

Fig. S7 UV–*vis* absorption spectra of **Pt8** on spin-coated film after different thermal annealing treatments.

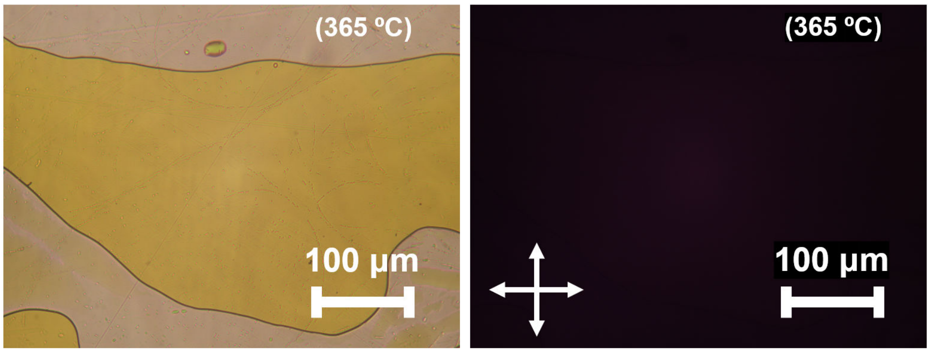

Fig. S8 POM images of **Pt8** at clear point temperature on heating.

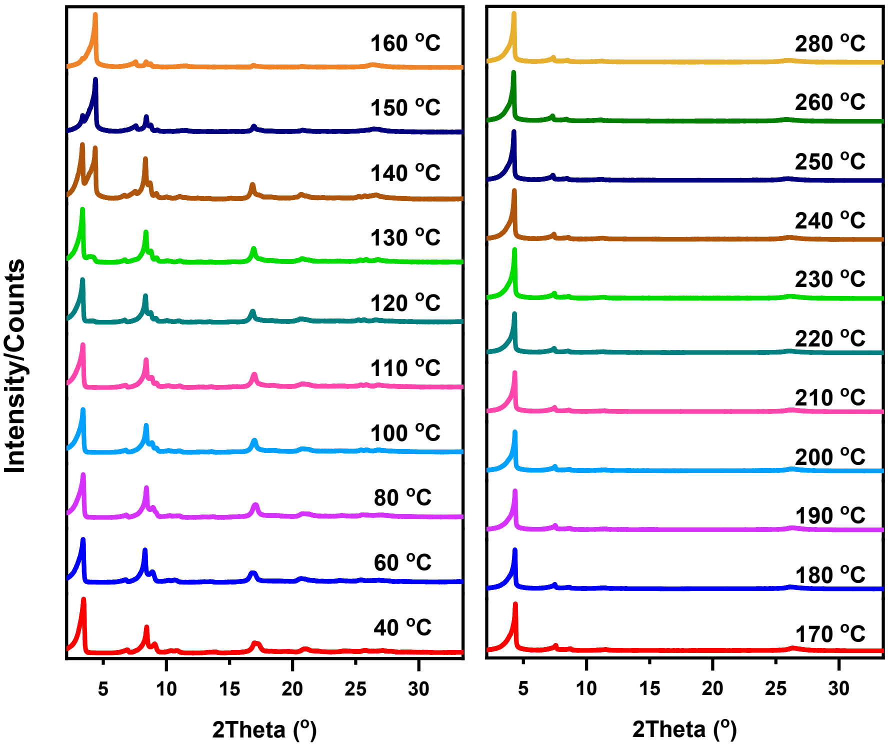

Fig. S9 XRD patterns of **Pt8** at different temperatures on cooling.

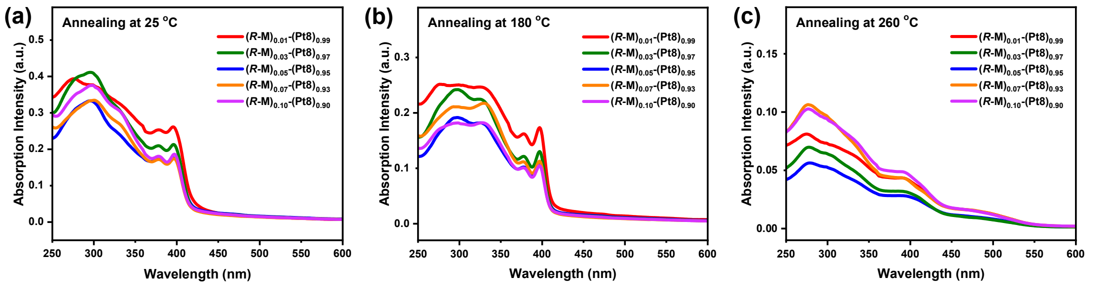

Fig. S10 UV−*vis* absorption spectra of ***R*-M-Pt8** on spin-coated film after different thermal annealing treatments.

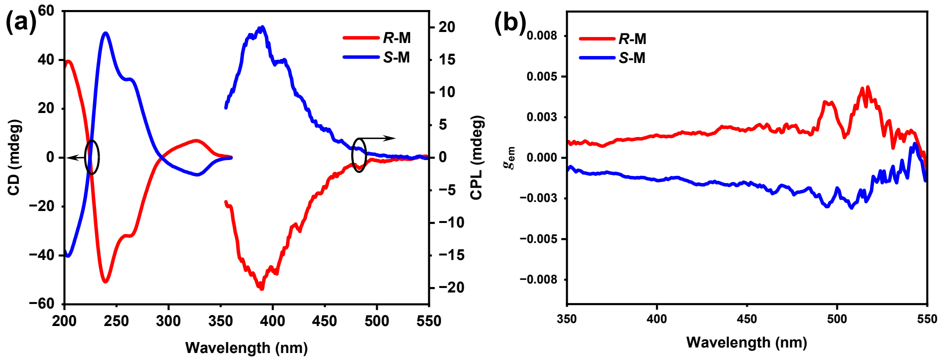

Fig. S11 CD and CPL spectra (a), and g_{em} values *vs* wavelength (b) of ***R*/*S*-M** on spin-coated film.

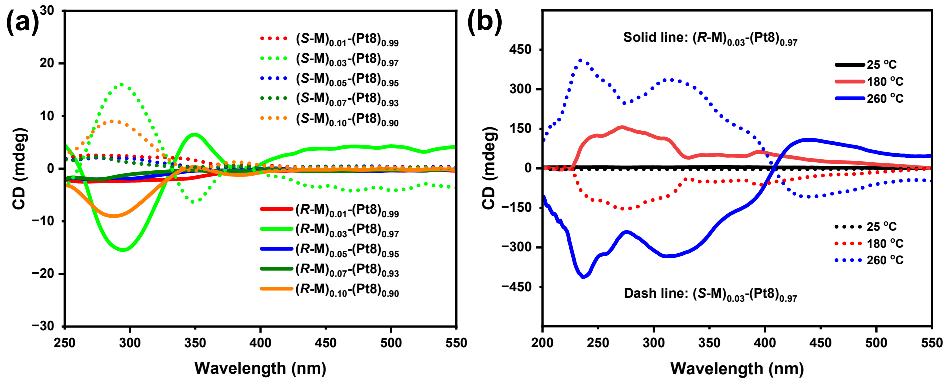

Fig. S12 CD spectra of spin-coated films ***R/S*-M-Pt8** at different molar ratios (a); CD spectra of **(*R/S*-M)$_{0.03}$-(Pt8)$_{0.97}$** at different annealing temperatures (b).

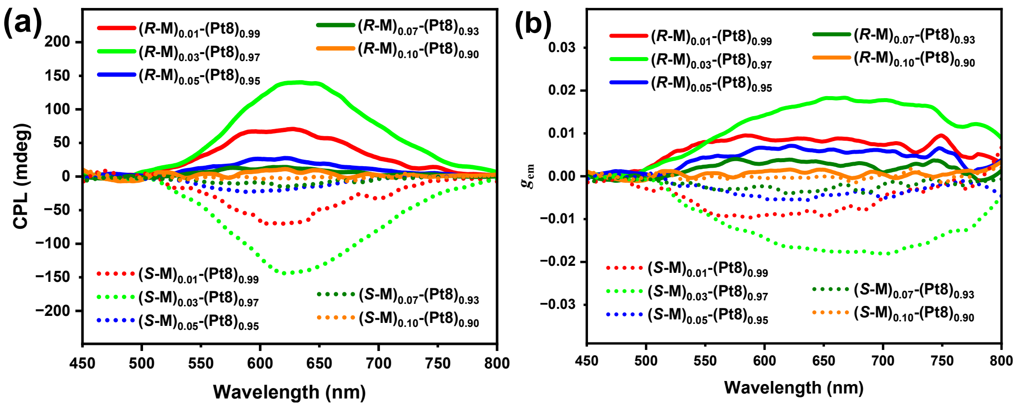

Fig. S13 CPL spectra (a) and g_{em} values *vs* wavelength (b) of spin-coated films ***R/S*-M-Pt8** at room temperature.

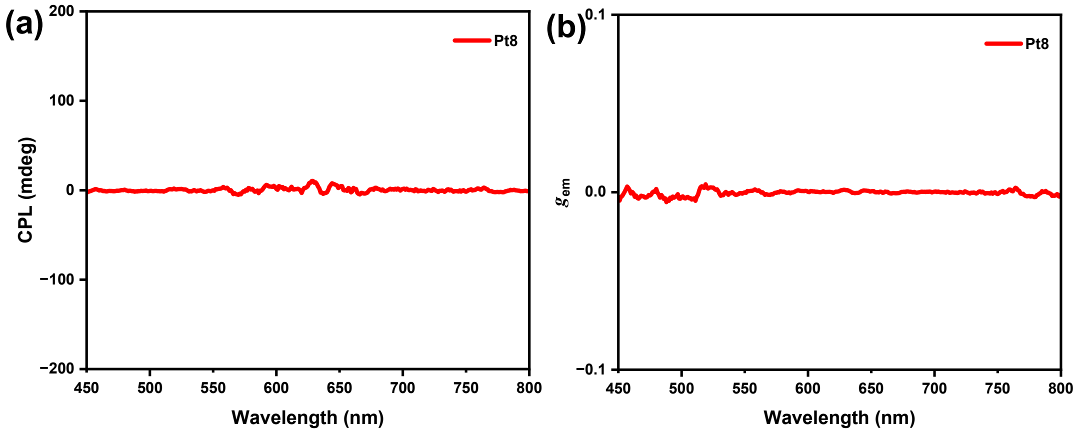

Fig. S14 CPL spectrum (a) and g_{em} values *vs* wavelength (b) of homoleptic Pt(II) complex **Pt8** on spin-coated film.

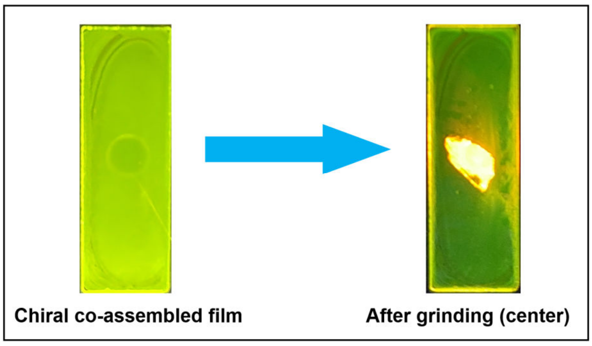

Fig. S15 The images of 180 °C-annealed film **(*R*-M)$_{0.03}$-(Pt8)$_{0.97}$** before and after grinding with a tweezer (at center).

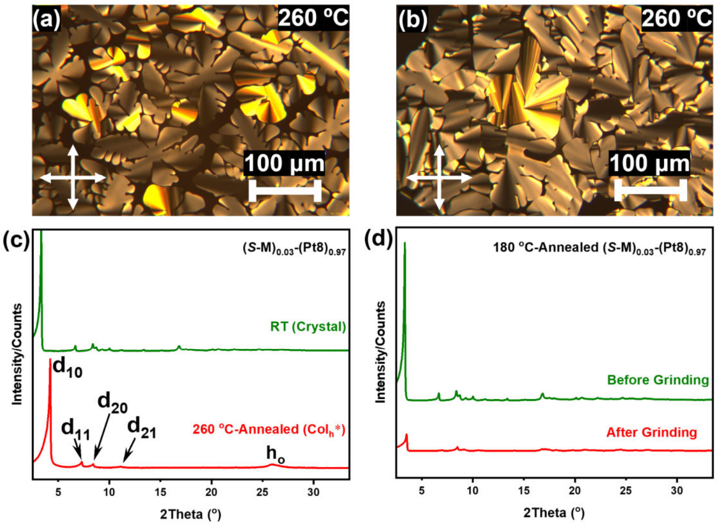

Fig. S16 POM images of **(*R*-M)0.03-(Pt8)0.97** (a) and **(*S*-M)0.03-(Pt8)0.97** (b); XRD patterns of **(*S*-M)0.03-(Pt8)0.97** at RT and 260 ºC (c); XRD patterns of 180 ºC-annealed **(*S*-M)0.03-(Pt8)0.97** before and after mechanical grinding (d).

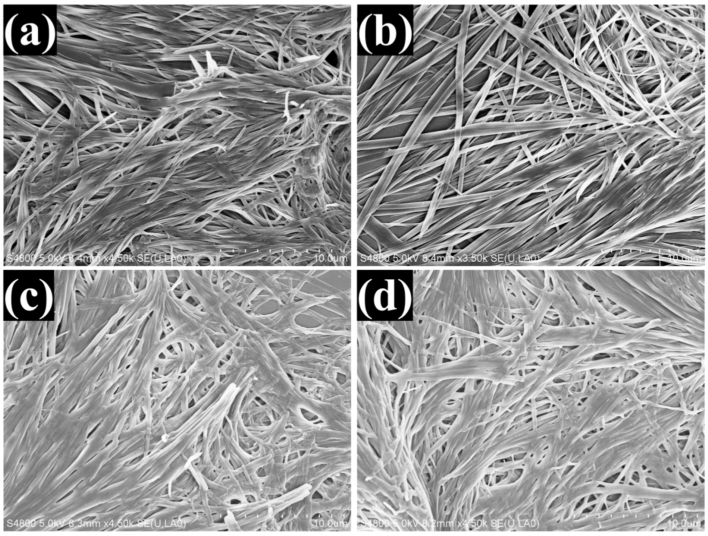

Fig. S17 SEM images of **(*R*-M)0.03-(Pt8)0.97** (a and c) and **(*S*-M)0.03-(Pt8)0.97** (b and d) in drop-casted films before (a and b) and after (c and d) thermal annealing at 180 ºC (a, b, c and d: film, 1.0×10^{-3} mol L^{-1} in chloroform).

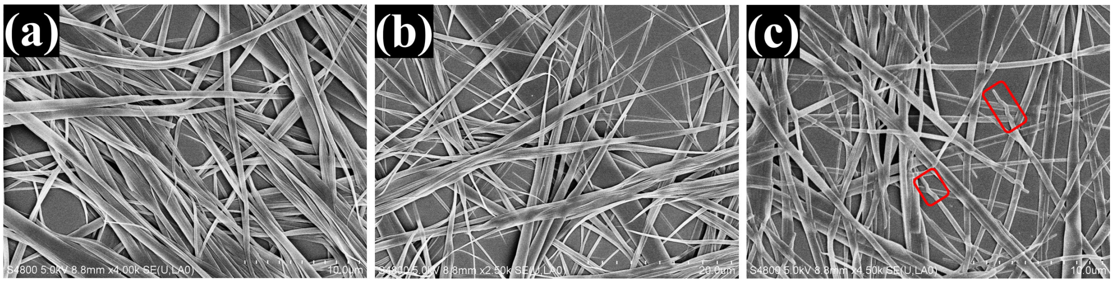

Fig. S18 SEM images of **(*R*-M)0.03-(Pt8)0.97** (a) and **(*S*-M)0.03-(Pt8)0.97** (b and c) in drop-casted films before (a and b) and after (c) thermal annealing at 180 ºC (a, b and c: film, 1.0×10^{-4} mol L^{-1} in chloroform).

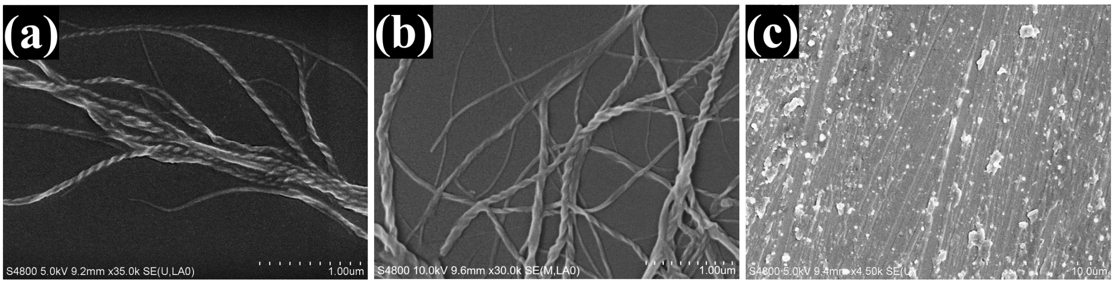

Fig. S19 SEM images of **(*R*-M)0.03-(Pt8)0.97** (a) and **(*S*-M)0.03-(Pt8)0.97** (b) in drop-casted films after thermal annealing at 260 ºC (a and b: film, 1.0 × 10−4 mol L−1 in chloroform). SEM image of 180 ºC-annealed **(*S*-M)0.03-(Pt8)0.97** after mechanical grinding (c).

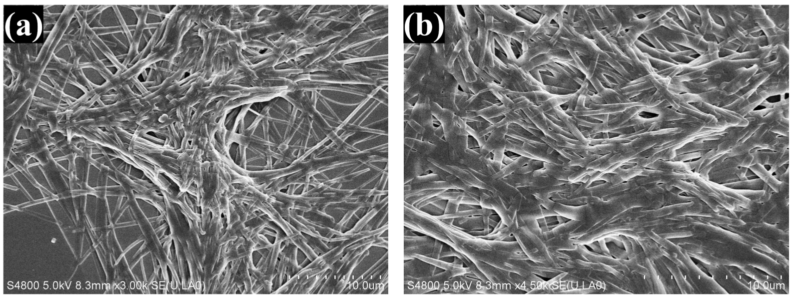

Fig. S20 SEM images of grinding films **(*R*-M)0.03-(Pt8)0.97** (a) and **(*S*-M)0.03-(Pt8)0.97** (b) after reheating to 180 ºC (film, 1.0 × 10−3 mol L−1 in chloroform).

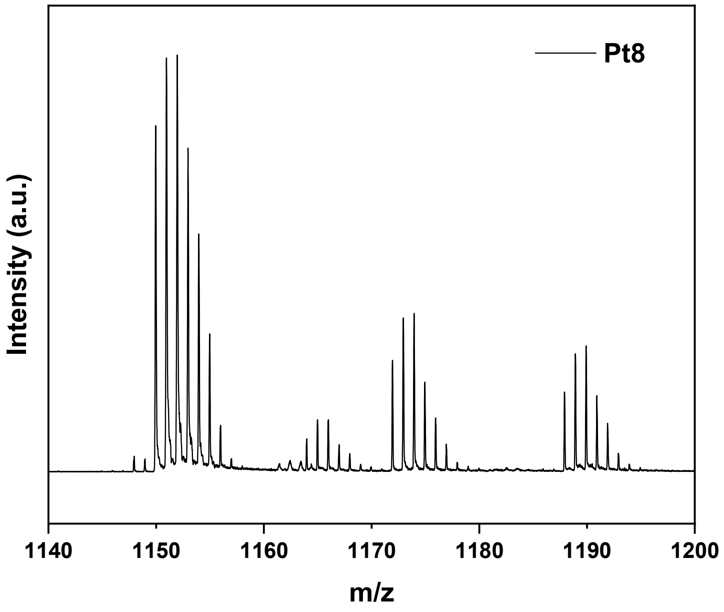

Fig. S21 MALDI-TOF MS spectrum of Pt(II) complex **Pt8**.

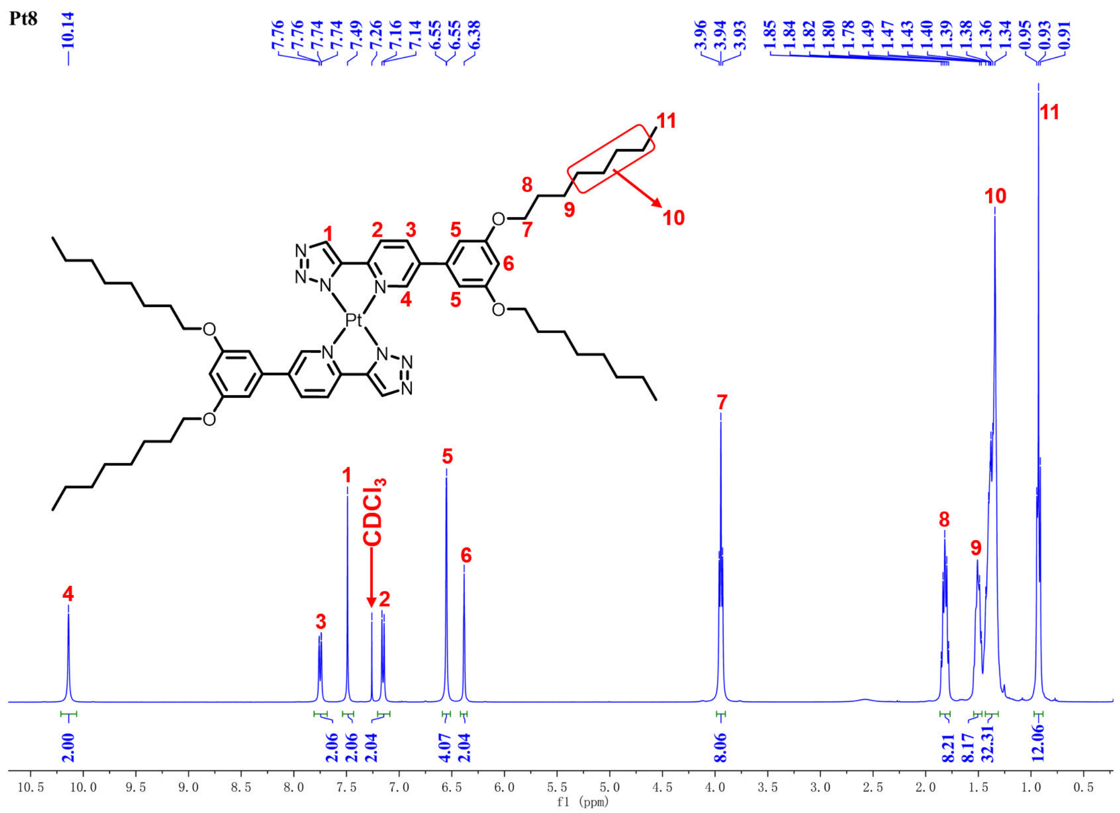

Fig. S22 ^{1}H NMR (400 MHz, $CDCl_3$) spectrum of Pt(II) complex **Pt8**.

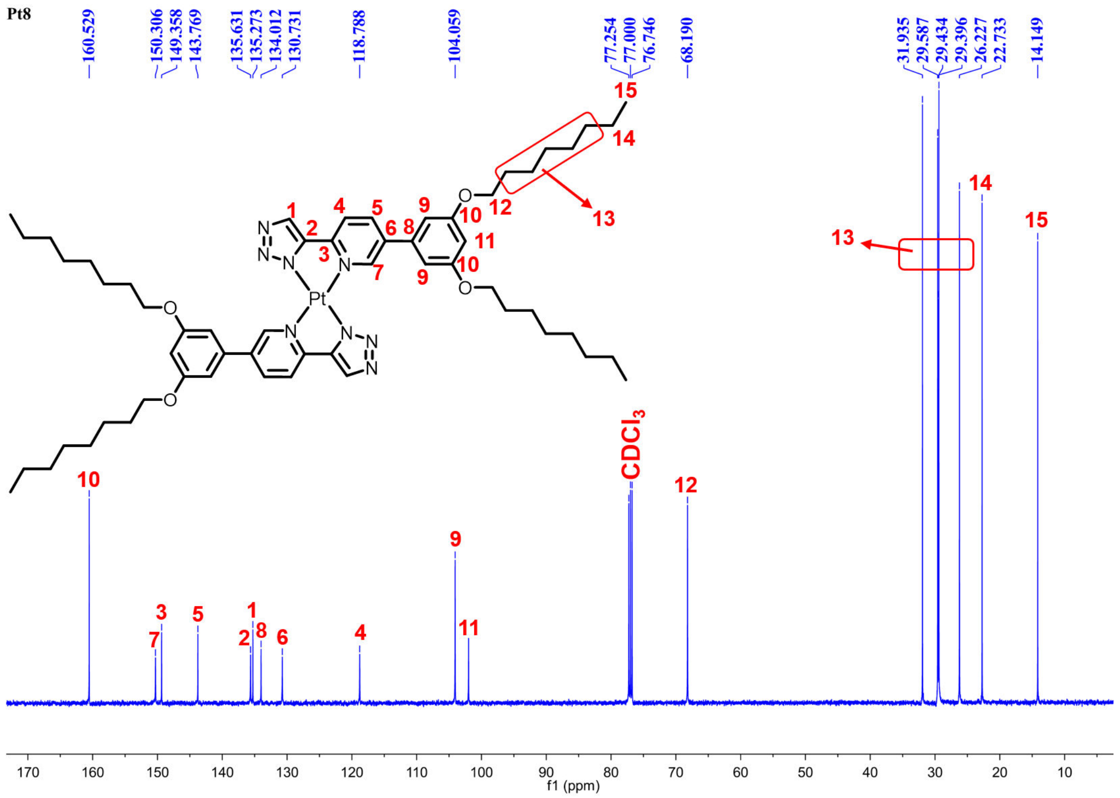

Fig. S23 ^{13}C NMR (126 MHz, $CDCl_3$) spectrum of Pt(II) complex **Pt8**.

5. References

(1) Tuesuwan, B.; Kerwin, S. M. 2-Alkynyl-N-propargyl Pyridinium Salts: Pyridinium-Based Heterocyclic Skipped Aza-Enediynes that Cleave DNA by Deoxyribosyl Hydrogen-Atom Abstraction and Guanine Oxidation. *Biochemistry-US* **2006**, *45*, 7265-7276.

(2) Ishiyama, T.; Murata, M.; Miyaura, N. Palladium(0)-Catalyzed Cross-Coupling Reaction of Alkoxydiboron with Haloarenes: A Direct Procedure for Arylboronic Esters. *J. Org. Chem.* **1995**, *60*, 7508-7510.

(3) Sharpless, K. B.; Loren, J. C.; Krasiński, A.; Fokin, V. V. NH-1,2,3-Triazoles from Azidomethyl Pivalate and Carbamates: Base-Labile N-Protecting Groups. *Synlett* **2005**, *9*, 2847-2850.

(4) Frisch, M. J. T., G. W.; Schlegel, H. B.; Scuseria, G. E.; Robb, M. A.; Cheeseman, J. R.; Scalmani, G.; Barone, V.; Petersson, G. A.; Nakatsuji, H.; Li, X.; Caricato, M.; Marenich, A. V.; Bloino, J.; Janesko, B. G.; Gomperts, R.; Mennucci, B.; Hratchian, H. P.; Ortiz, J. V.; Izmaylov, A. F.; Sonnenberg, J. L.; Williams, Ding, F.; Lipparini, F.; Egidi, F.; Goings, J.; Peng, B.; Petrone, A.; Henderson, T.; Ranasinghe, D.; Zakrzewski, V. G.; Gao, J.; Rega, N.; Zheng, G.; Liang, W.; Hada, M.; Ehara, M.; Toyota, K.; Fukuda, R.; Hasegawa, J.; Ishida, M.; Nakajima, T.; Honda, Y.; Kitao, O.; Nakai, H.; Vreven, T.; Throssell, K.; Montgomery Jr., J. A.; Peralta, J. E.; Ogliaro, F.; Bearpark, M. J.; Heyd, J. J.; Brothers, E. N.; Kudin, K. N.; Staroverov, V. N.; Keith, T. A.; Kobayashi, R.; Normand, J.; Raghavachari, K.; Rendell, A. P.; Burant, J. C.; Iyengar, S. S.; Tomasi, J.; Cossi, M.; Millam, J. M.; Klene, M.; Adamo, C.; Cammi, R.; Ochterski, J. W.; Martin, R. L.; Morokuma, K.; Farkas, O.; Foresman, J. B.; Fox, D. J. *Gaussian 16, Revision B.01 Gaussian, Inc., Wallingford CT,: 2016*,

(5) Stephens, P. J.; Devlin, F. J.; Chabalowski, C. F.; Frisch, M. J. Ab Initio Calculation of Vibrational Absorption and Circular Dichroism Spectra Using Density Functional Force Fields. *J. Phys. Chem.* **1994**, *98*, 11623-11627.

(6) Marenich, A. V.; Cramer, C. J.; Truhlar, D. G. Universal Solvation Model Based on Solute Electron Density and on a Continuum Model of the Solvent Defined by the Bulk Dielectric Constant and Atomic Surface Tensions. *J. Phys. Chem. B* **2009**, *113*, 6378-6396.

(7) Lu, T.; Chen, F. Multiwfn: A multifunctional wavefunction analyzer. *J. Comput. Chem.* **2012**, *33*, 580-592.

(8) Grimme, S.; Antony, J.; Ehrlich, S.; Krieg, H. A consistent and accurate ab initio parametrization of density functional dispersion correction (DFT-D) for the 94 elements H-Pu. *J. Chem. Phys* **2010**, *132*, 154104.

(9) Johnson, E. R.; Becke, A. D. A post-Hartree–Fock model of intermolecular interactions. *J. Chem. Phys* **2005**, *123*, 024101.

(10) Becke, A. D.; Johnson, E. R. A density-functional model of the dispersion interaction. *J. Chem. Phys* **2005**, *123*, 154101.

(11) Johnson, E. R.; Becke, A. D. A post-Hartree-Fock model of intermolecular interactions: Inclusion of higher-order corrections. *J. Chem. Phys* **2006**, *124*, 174104.

(12) Grimme, S.; Ehrlich, S.; Goerigk, L. Effect of the damping function in dispersion corrected density functional theory. *J. Comput. Chem.* **2011**, *32*, 1456-1465.

(13) Prabhath, M. R. R.; Romanova, J.; Curry, R. J.; Silva, S. R. P.; Jarowski, P. D. The Role of Substituent Effects in Tuning Metallophilic Interactions and Emission Energy of Bis-4-(2-pyridyl)-1,2,3-triazolatoplatinum(II) Complexes. *Angew. Chem. Int. Ed.* **2015**, *54*, 7949-7953.

(14) Zou, G.; Zhao, L.; Zeng, L.; Luo, K.; Ni, H.; Wang, H.; Li, Q.; Yu, W.; Li, X. Columnar Iridium(III)

Metallomesogens Based on Polycatenar Pyridyltetrazolate with Ambipolar Carrier Mobility Behavior. *Inorg. Chem.* **2019**, *58*, 861-869.

(15) Wu, J.; Watson, M. D.; Zhang, L.; Wang, Z.; Müllen, K. Hexakis(4-iodophenyl)-peri-hexabenzocoronene- A Versatile Building Block for Highly Ordered Discotic Liquid Crystalline Materials. *J. Am. Chem. Soc.* **2004**, *126*, 177-186.

(16) Yasuda, T.; Shimizu, T.; Liu, F.; Ungar, G.; Kato, T. Electro-Functional Octupolar π-Conjugated Columnar Liquid Crystals. *J. Am. Chem. Soc.* **2011**, *133*, 13437-13444.

(17) Jasiński, M.; Szymańska, K.; Gardias, A.; Pociecha, D.; Monobe, H.; Szczytko, J.; Kaszyński, P. Tuning the Magnetic Properties of Columnar Benzo[e][1,2,4]triazin-4-yls with the Molecular Shape. *ChemPhysChem* **2019**, *20*, 636-644.

(18) Zou, G.; Jiang, Z.; Li, D.; Li, Q.; Cheng, Y. Efficient helical columnar emitters of chiral homoleptic Pt(ii) metallomesogens for circularly polarized electroluminescence. *Chem. Sci.* **2024**, *15*, 18534-18542.

(19) Li, Y.; Urbas, A.; Li, Q. Reversible Light-Directed Red, Green, and Blue Reflection with Thermal Stability Enabled by a Self-Organized Helical Superstructure. *J. Am. Chem. Soc.* **2012**, *134*, 9573-9576.
